# Supplementary material for: Structural optimization of siRNA conjugates for albumin binding achieves effective MCL1-directed cancer therapy
Source: Nat Commun. 2024 Feb 21;15:1581. doi: 10.1038/s41467-024-45609-0 (PMC10881965; doi:10.1038/s41467-024-45609-0)
Supplement: Supplementary file 1 — Supplementary Information [file 41467_2024_45609_MOESM1_ESM.pdf]

Structural Optimization of siRNA Conjugates for Albumin Binding Achieves Effective MCL1-Directed Cancer Therapy

Ella N. Hoogenboezem[1], Shrusti S. Patel[1], Justin H. Lo[1,2], Ashley B. Cavnar[2], Lauren M. Babb[1], Nora Francini[1], Eva F. Gbur[1], Prarthana Patil[1], Juan M. Colazo[1,3], Danielle L. Michell[2], Violeta M. Sanchez[2], Joshua T. McCune[1], Jinqi Ma[1], Carlisle R. DeJulius[1], Linus H. Lee, Jonah C. Rosch[4], Ryan M. Allen[2], Larry D. Stokes[1], Jordan L. Hill[1], Kasey C. Vickers[2,5], Rebecca S. Cook[1], and Craig L. Duvall[1*]

1. Department of Biomedical Engineering, Vanderbilt University, Nashville, TN
2. Department of Medicine, Vanderbilt University Medical Center, Nashville, TN
3. Medical Scientist Training Program, Vanderbilt University School of Medicine, Nashville, TN
4. Department of Chemical and Biomolecular Engineering, Vanderbilt University, Nashville, TN
5. Department of Molecular Physiology and Biophysics, Vanderbilt University, Nashville, TN

Supplementary Information

Supplementary Methods

Synthesis of siRNA conjugate variant covalently bound to albumin

Conjugate covalently bound to albumin was synthesized by first reacting azido-PEG_3-maleimide (Click Chemistry Tools) with the free thiols on human (1 free SH) or mouse (2 free SH). Albumin was dissolved in PBS with 0.5M EDTA to a final concentration of 10 mM. Anhydrous DMF was used to solubilize and activate azido-PEG_3-maleimide. DBCO-modified siRNA duplex in PBS was reacted at a 1:1 ratio of DBCO groups:free SH groups and allowed to incubate at room temperature for 4 hours. To remove any siRNA that did not react with albumin, or reacted only with the azido linker, the resulting solution underwent 10 rounds of centrifugation in a 30 kDa cutoff Amicon filter at 14,000 xg for 10 minutes for each round. Conjugation was confirmed by gel electrophoresis of precusor DBCO-siRNA alongside resulting siRNA-DBCO-albumin.

Synthesis of PFP-modified lipids

Oleic acid was dissolved at 1mM in anhydrous dichloromethane and placed on ice. A 10x molar excess of triethylamine was then added followed by a 3x molar excess of pentafluorophenyl trifluoroacetate. A similar procedure was used for octadecanoic acid. The lipid was dissolved at 0.5 mM in anhydrous dichloromethane and placed on ice. A 2x molar excess of triethylamine was then added followed by a 0.5x molar excess of pentafluorophenyl trifluoroacetate added dropwise.

For both lipids, the reaction vessel was removed from ice after 15 minutes and allowed to equilibrate to room temperature. The reaction mixture was then stirred for 4h followed by storage at -20C. The crude product was purified by silica gel column chromatography and eluted with solvents with a gradient from 100% hexane to 100% ethyl acetate to yield compounds 1 and 2. The compounds were then rotary evaporated to dryness and characterized by ^{1}H- and ^{19}F-NMR spectroscopy.

Synthesis of lipid-variant siRNA conjugates

Amine-terminated oligonucleotides were speed vacuumed to dryness and desalted to remove MMT groups. Oligonucleotides were then lyophilized followed by reconstitution in 0.1 sodium tetraborate (pH 8.5) to a concentration of 500 μM. PFP-modified lipid was dissolved into a mixture of acetonitrile, DMSO, and triethylamine (70:29:1 by volume) at a concentration of 7 μM. Aqueous oligonucleotide was added dropwise to the organic solution for a 1:40 molar ratio of oligonucleotide-amine:amine-reactive lipid (approximately 25% 0.1M sodium tetraborate, 75% organic mixture). The solution was stirred overnight and desalted prior to purification and characterization, as detailed in the main manuscript methods section.

Supplementary Table 1: siRNA sequence and modification details

	**Sequence (5' to 3')**
Luciferase (Luc) Antisense	(PHO) (OMeU)*(fU)*(OMeC) (fA) (OMeU) (fU) (OMeA) (fU) (OMeC) (fA) (OMeG) (fU) (OMeG) (fC) (OMeA) (fA) (OMeU)*(fU)*(OMeG)
Luc Sense	(fC)*(OMeA)*(fA) (OMeU) (fU) (OMeG) (fC) (OMeA) (fC) (OMeU) (fG) (OMeA) (fU) (OMeA) (fA) (OMeU) (fG)*(OMeA)*(fA)
Scrambled (Scr) Antisense	(PHO) (OMeG)*(fU)*(OMeA) (fU) (OMeU) (fA) (OMeU) (fA) (OMeC) (fG) (OMeC) (fG) (OMeA) (fU) (OMeU) (fA) (OMeA) (fC) (OMeG)*(fA)*(OMeC)
Scr Sense	(fC)*(OMeG)*(fU) (OMeU) (fA) (OMeA) (fU) (OMeC) (fG) (OMeC) (fG) (OMeU) (fA) (OMeU) (fA) (OMeA) (fU)*(OMeA)*(fC)
Cy5-labeled Luc Antisense	(Cy5) (OMeU)*(fU)*(OMeC) (fA) (OMeU) (fU) (OMeA) (fU) (OMeC) (fA) (OMeG) (fU) (OMeG) (fC) (OMeA) (fA) (OMeU)*(fU)*(OMeG)
Biotin-TEG-labeled Luc Antisense	(BTN-TEG)*(OMeU)*(fU)*(OMeC) (fA) (OMeU) (fU) (OMeA) (fU) (OMeC) (fA) (OMeG) (fU) (OMeG) (fC) (OMeA) (fA) (OMeU)*(fU)*(OMeG)
Mcl-1 Antisense	(fC)*(OMeA)*(fU)(OMeC)(fG)(OMeA)(fA)(OMeC)(fC)(OMeA)(fU)(OMeU)(fA)(OMeG)(fC)(OMeA)(fG)*(OMeA)*(fA)
Mcl-1 Sense	(PHO)(OMeU)*(fU)*(OMeC) (fU)(OMeG)(fC) (OMeU)(fA)(OMeA) (fU)(OMeG)(fG) (OMeU)(fU)(OMeC) (fG)(OMeA)*(fU)*(OMeG)
Phosphorothioate bond (X)(X)*<br>*Phosphodiester bond (X) (X)*<br>*2'F substituted base (fX)*<br>*2'OMe substituted base (OMeX)*	

Supplementary Table 2: Pharmacokinetic parameters for full siRNA conjugate library determined from intravital microscopy

		$t_{1/2}$ (min)	$AUC_{0\text{-}35m}$ (µg·min)/(mL)	CL (mL)/(min)
	siRNA	14 ± 3.5	210 ± 25	0.069 ± 0.012
	si-EG$_3$-cholesterol	28 ± 12	410 ± 42	0.025 ± 0.0079
	si-PEG$_{45}$<L$_2$	27 ± 5.3	390 ± 25	0.026 ± 0.0046
EG Variants	si<(EG$_0$L)$_2$	33 ± 6.6	416 ± 55	0.028 ± 0.0059
EG Variants	si<(EG$_6$L)$_2$	36 ± 4.6	450 ± 15	0.019 ± 0.0022
EG Variants	si<(EG$_{18}$L)$_2$	64 ± 23	470 ± 52	0.012 ± 0.0032
EG Variants	si<(EG$_{30}$L)$_2$	37 ± 6.6	440 ± 36	0.019 ± 0.0043
PS Variants	si<(EG$_{18}$L)$_2$ No 5’Se PS	37 ± 12	430 ± 50	0.020 ± 0.0060
PS Variants	si<(EG$_{18}$L)$_2$ No 5’Se or Binder PS	15 ± 1.5	300 ± 36	0.047 ± 0.0070
Branching Variants	si-(EG$_{18}$)<L$_2$	67 ± 32	490 ± 35	0.012 ± 0.0070
Branching Variants	si-(EG$_{36}$)<L$_2$	41 ± 13	450 ± 27	0.018 ± 0.0050
Lipid Variants	si<(EG$_{18}$L$_{diacid}$)$_2$	47 ± 21	469 ± 64	0.017 ± 0.0073
Lipid Variants	si<(EG$_{18}$L$_{unsaturated}$)$_2$	34 ± 26	391 ± 121	0.029 ± 0.016

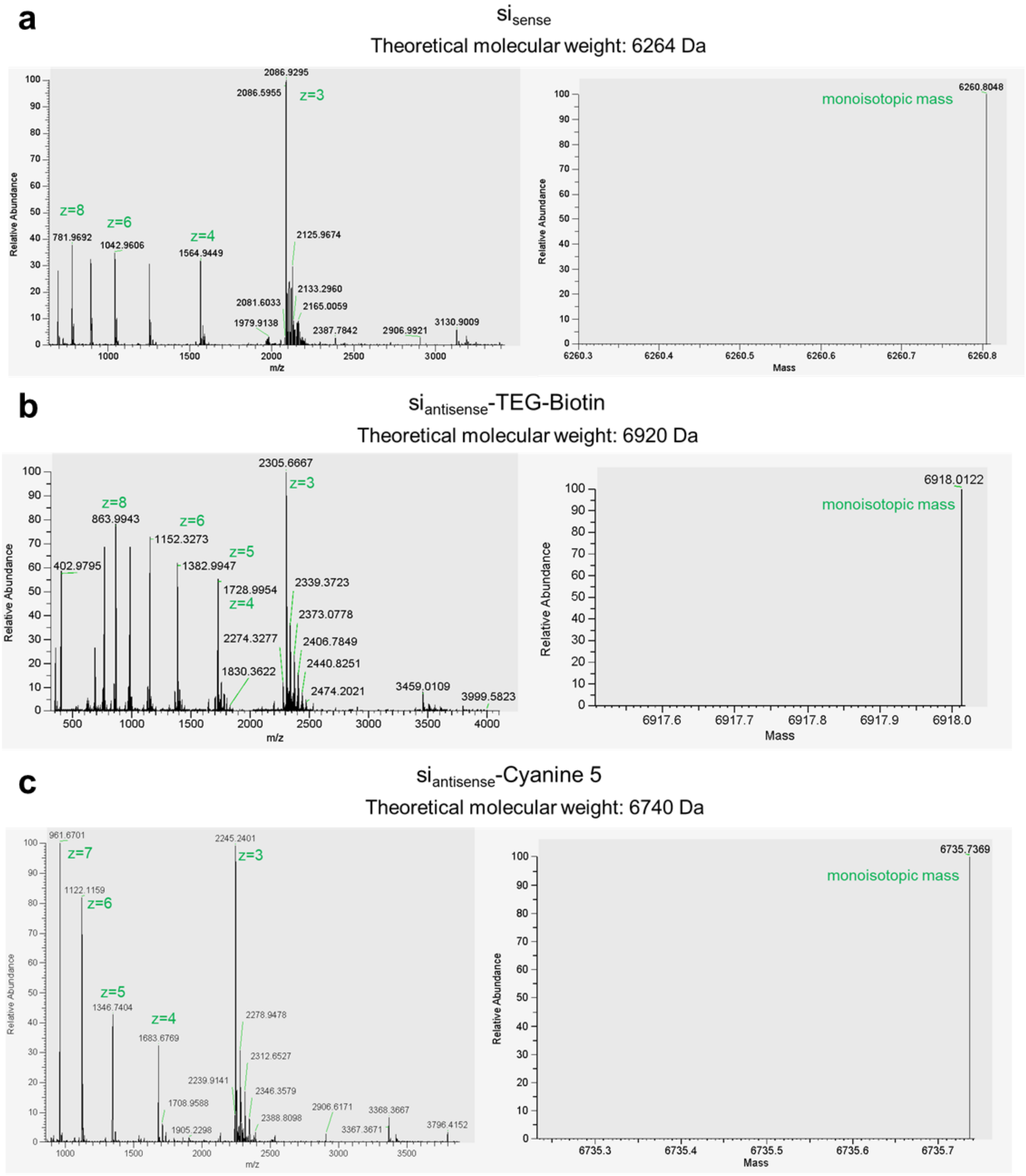
a
si sense
Theoretical molecular weight: 6264 Da
Relative Abundance
m/z
z=3
z=8
z=6
z=4
2086.9295
2086.5955
781.9692
1042.9606
1564.9449
2125.9674
2133.2960
2081.6033
2165.0059
1979.9138
2387.7842
2906.9921
3130.9009
monoisotopic mass
6260.8048
Mass
b
si antisense-TEG-Biotin
Theoretical molecular weight: 6920 Da
Relative Abundance
m/z
2305.6667
z=3
z=8
863.9943
z=6
1152.3273
z=5
402.9795
1382.9947
1728.9954
z=4
2339.3723
2373.0778
2274.3277
2406.7849
2440.8251
1830.3622
2474.2021
3459.0109
3999.5823
monoisotopic mass
6918.0122
Mass
c
si antisense-Cyanine 5
Theoretical molecular weight: 6740 Da
Relative Abundance
m/z
961.6701
z=7
1122.1159
z=6
2245.2401
z=3
z=5
1346.7404
z=4
1683.6769
2278.9478
2312.6527
2239.9141
2346.3579
1708.9588
2388.8098
1905.2298
2906.6171
3367.3671
3368.3667
3796.4152
monoisotopic mass
6735.7369
Mass

d

si-L_1

Theoretical molecular weight: 6612 Da

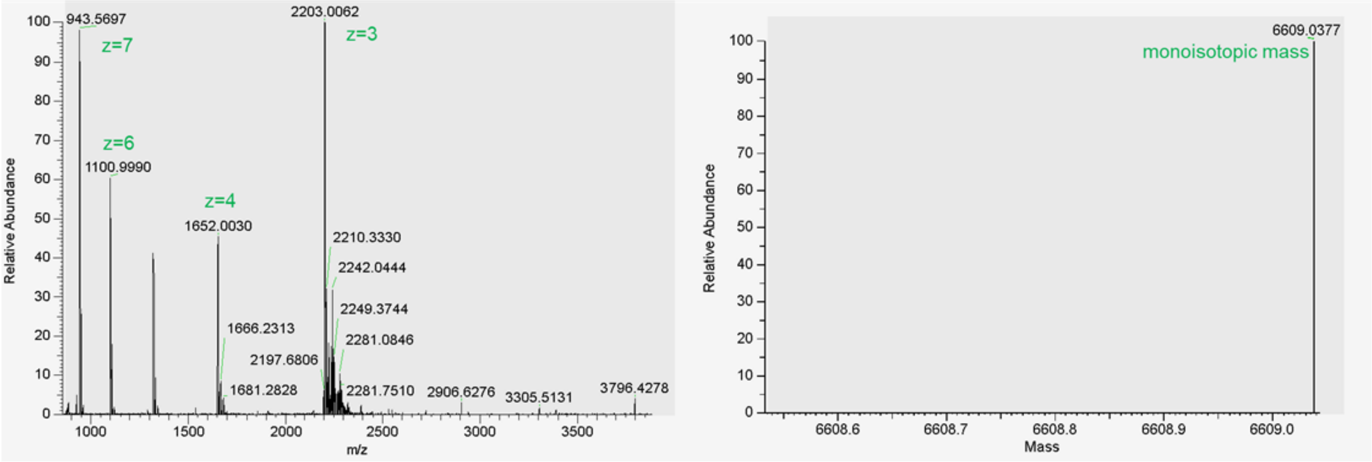

e

si-EG_3-chol

Theoretical molecular weight: 6963 Da

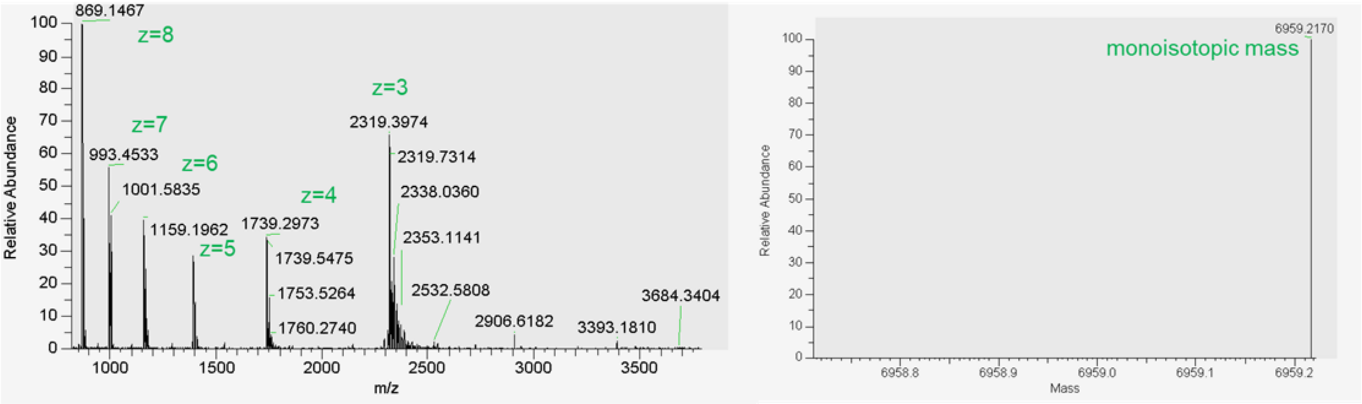

f

si<$(EG_0L)_2$

Theoretical molecular weight: 7126 Da

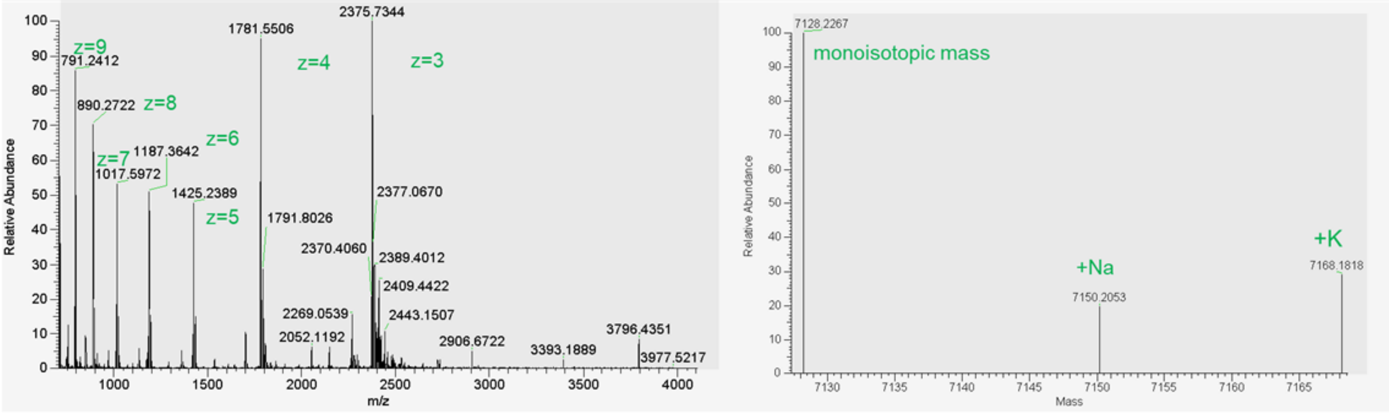

g

$si<(EG_6L)_2$

Theoretical molecular weight: 7846 Da

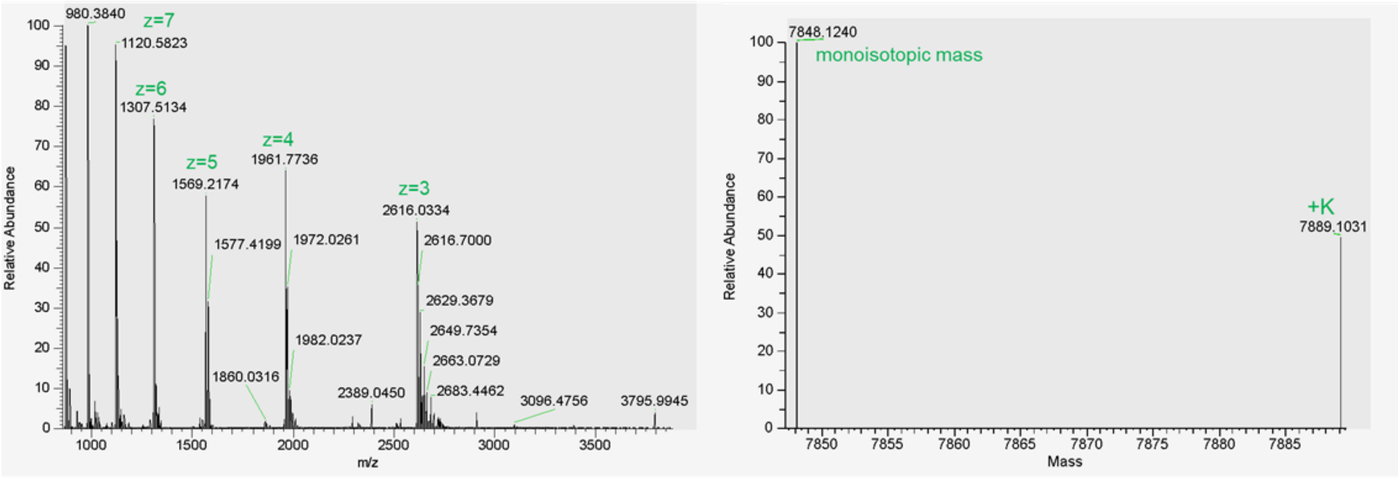

h

$si<(EG_6L)_2$

Theoretical molecular weight: 7846 Da

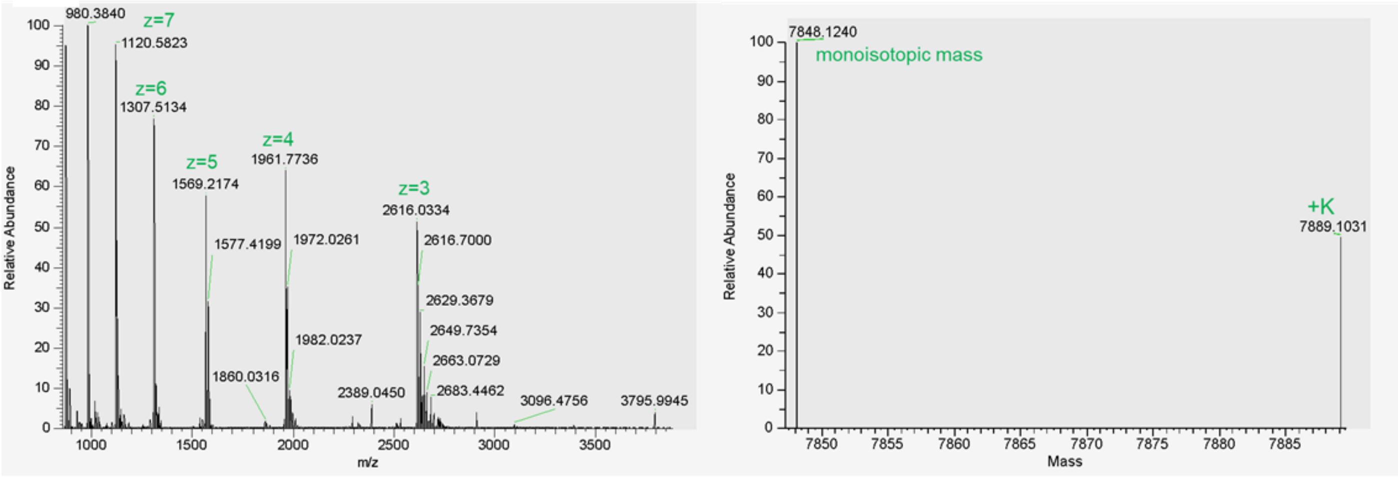

i

$si<(EG_{30}L)_2$

Theoretical molecular weight: 10,735 Da

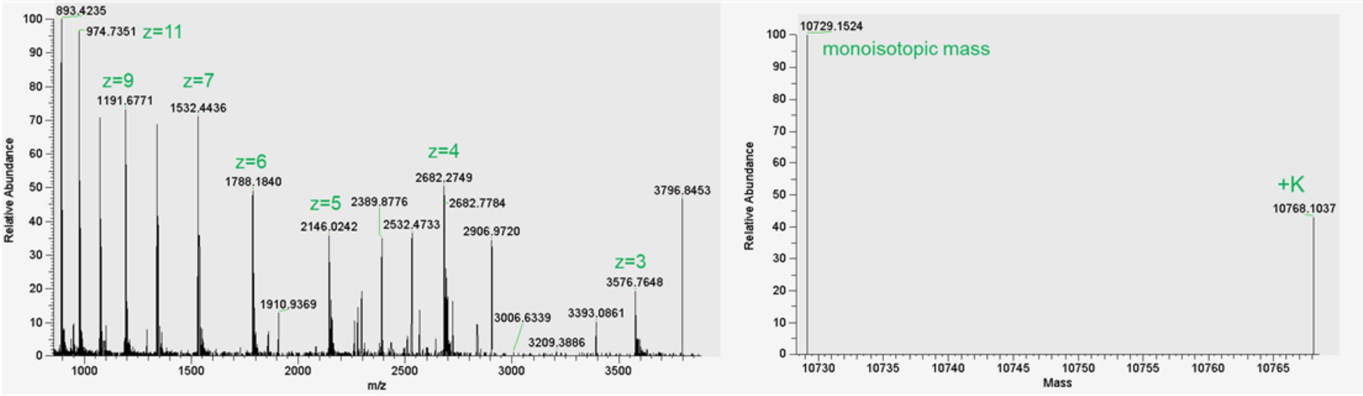

j

si<(EG$_{18}$L)$_2$No 5' PS or Binder PS

Theoretical molecular weight: 9115 Da

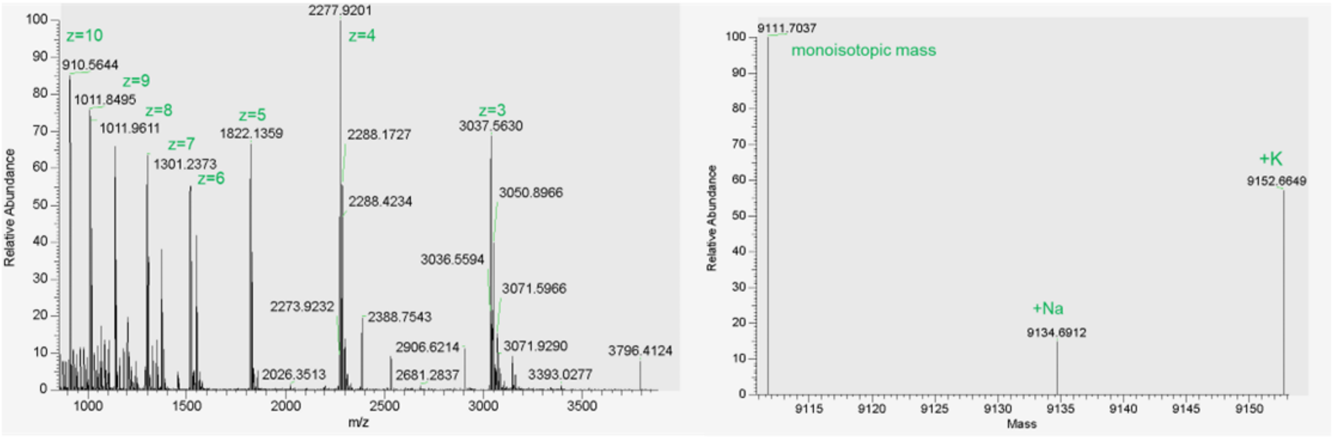

k

si<(EG$_{18}$L)$_2$No 5' PS

Theoretical molecular weight: 9260 Da

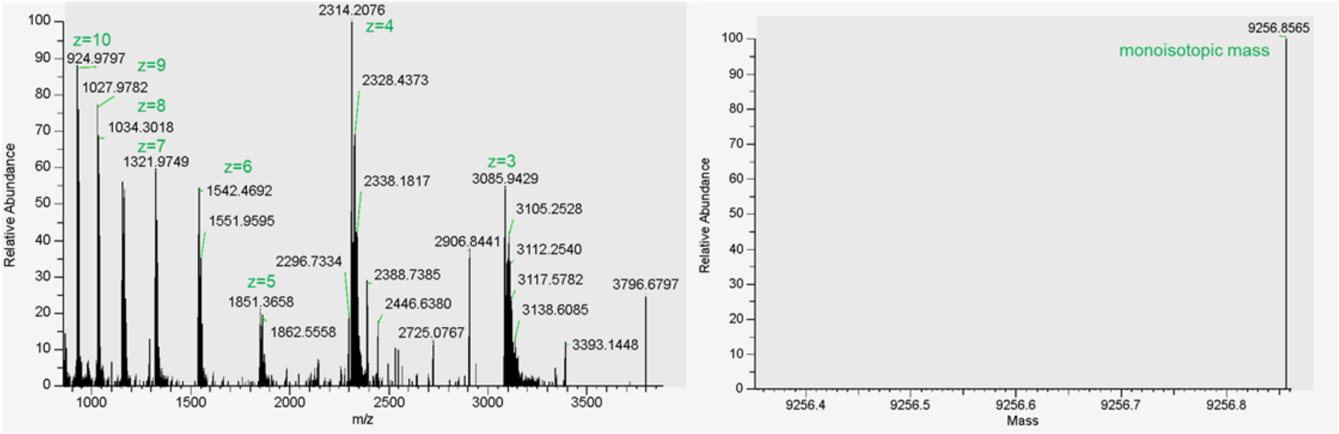

l

si-EG$_{18}$<L$_2$

Theoretical molecular weight: 8211 Da

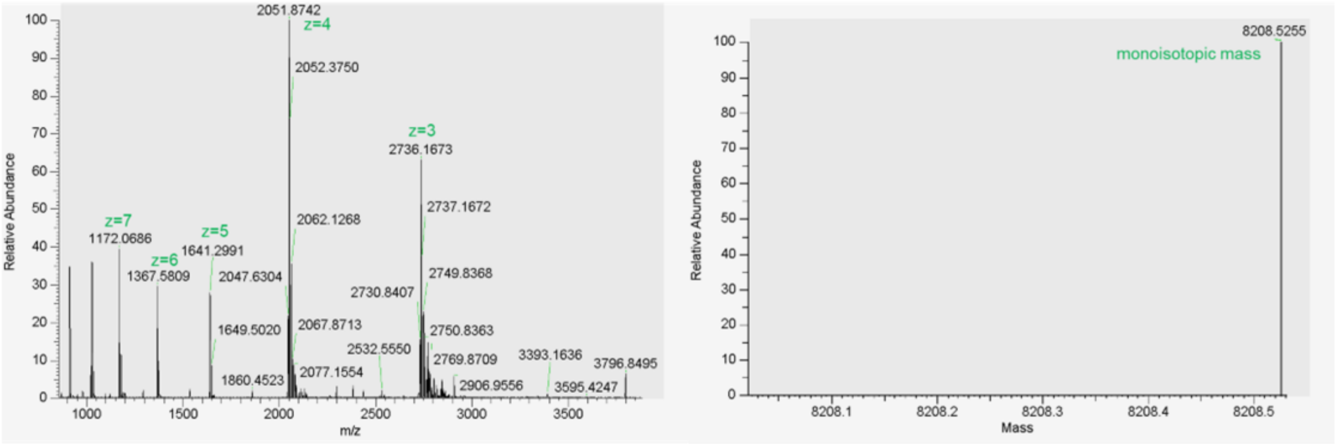

m

si-EG$_{36}$<L$_2$
Theoretical molecular weight: 9292 Da

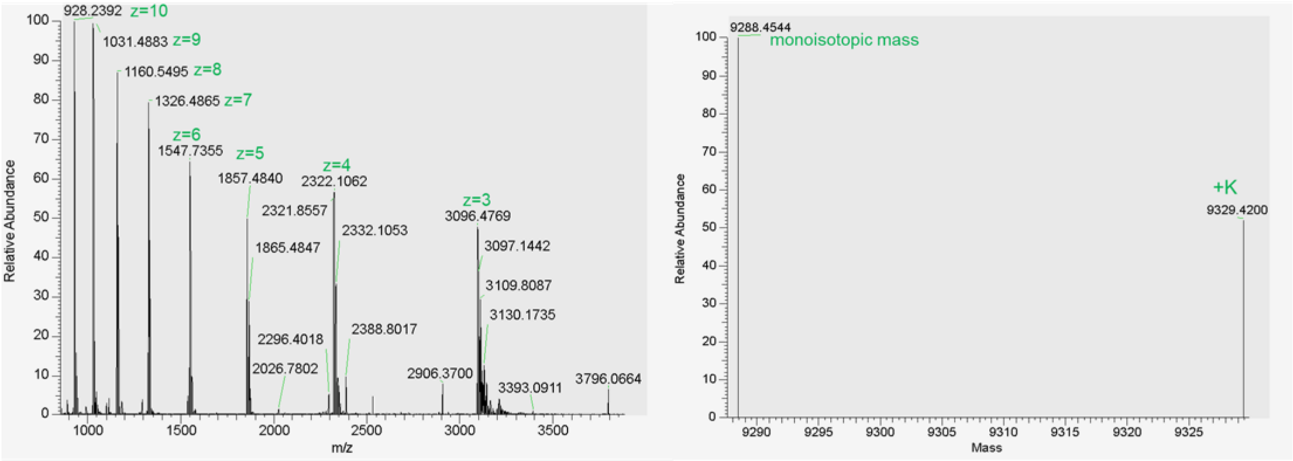

n

si<(EG$_{18}$L$_{unsaturated}$)$_2$
Target molecular weight 9490

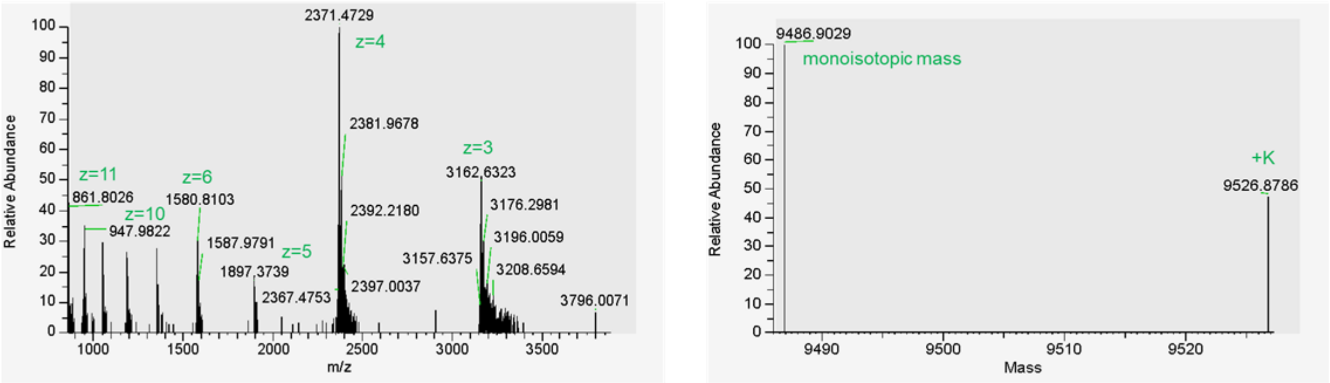

o

si<(EG$_{18}$L$_{diacid}$)$_2$
Target molecular weight 9554

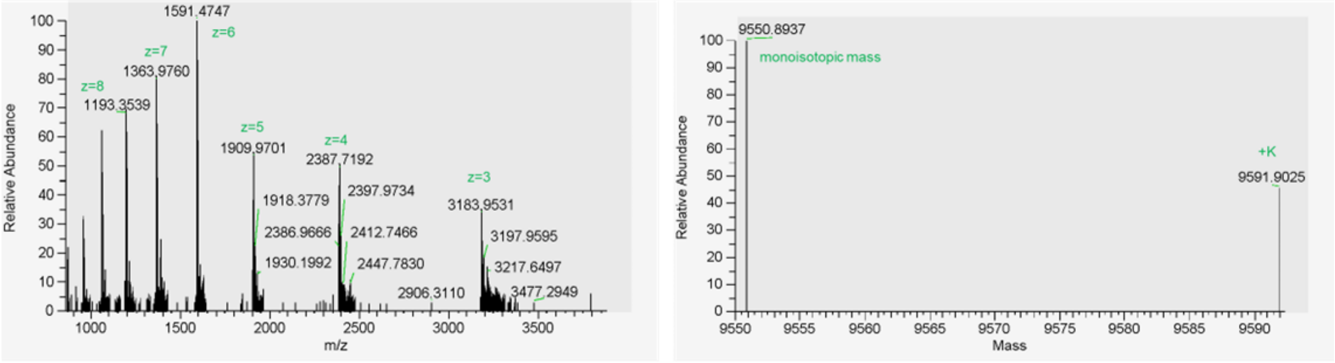

p

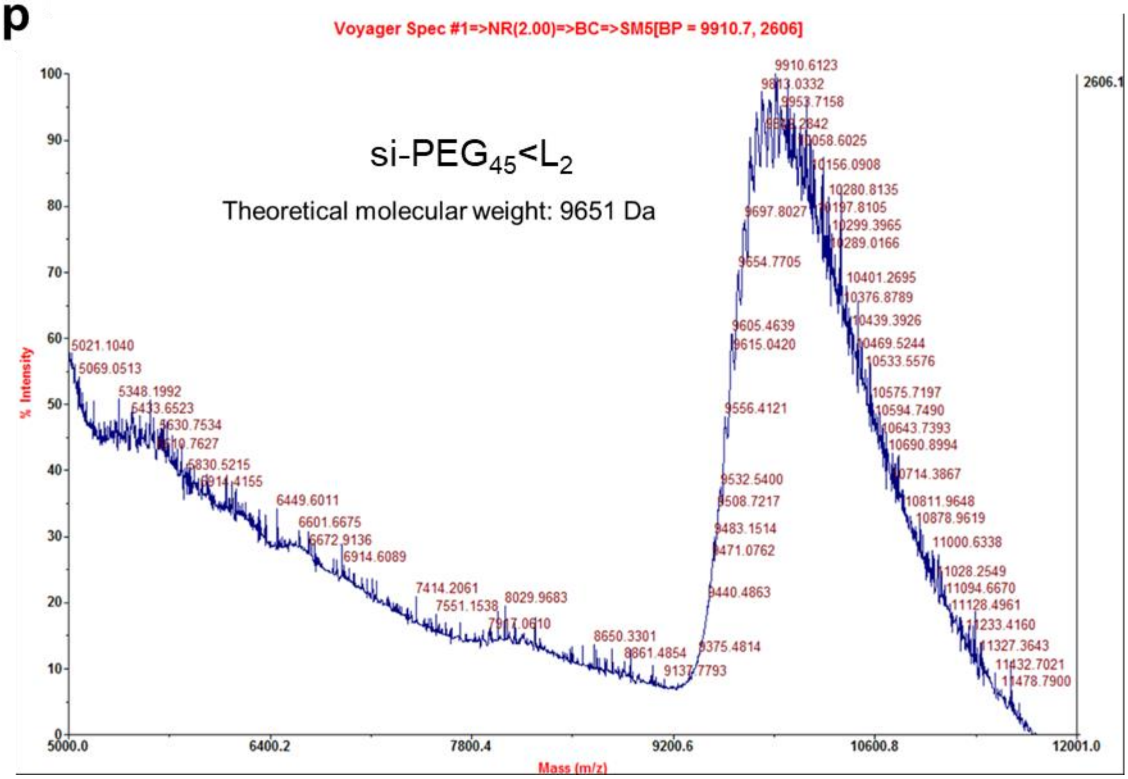
Voyager Spec #1=>NR(2.00)=>BC=>SM5[BP = 9910.7, 2606]
si-PEG45<L2
Theoretical molecular weight: 9651 Da
% Intensity
Mass (m/z)
2606.1
9910.6123
5021.1040
11478.7900

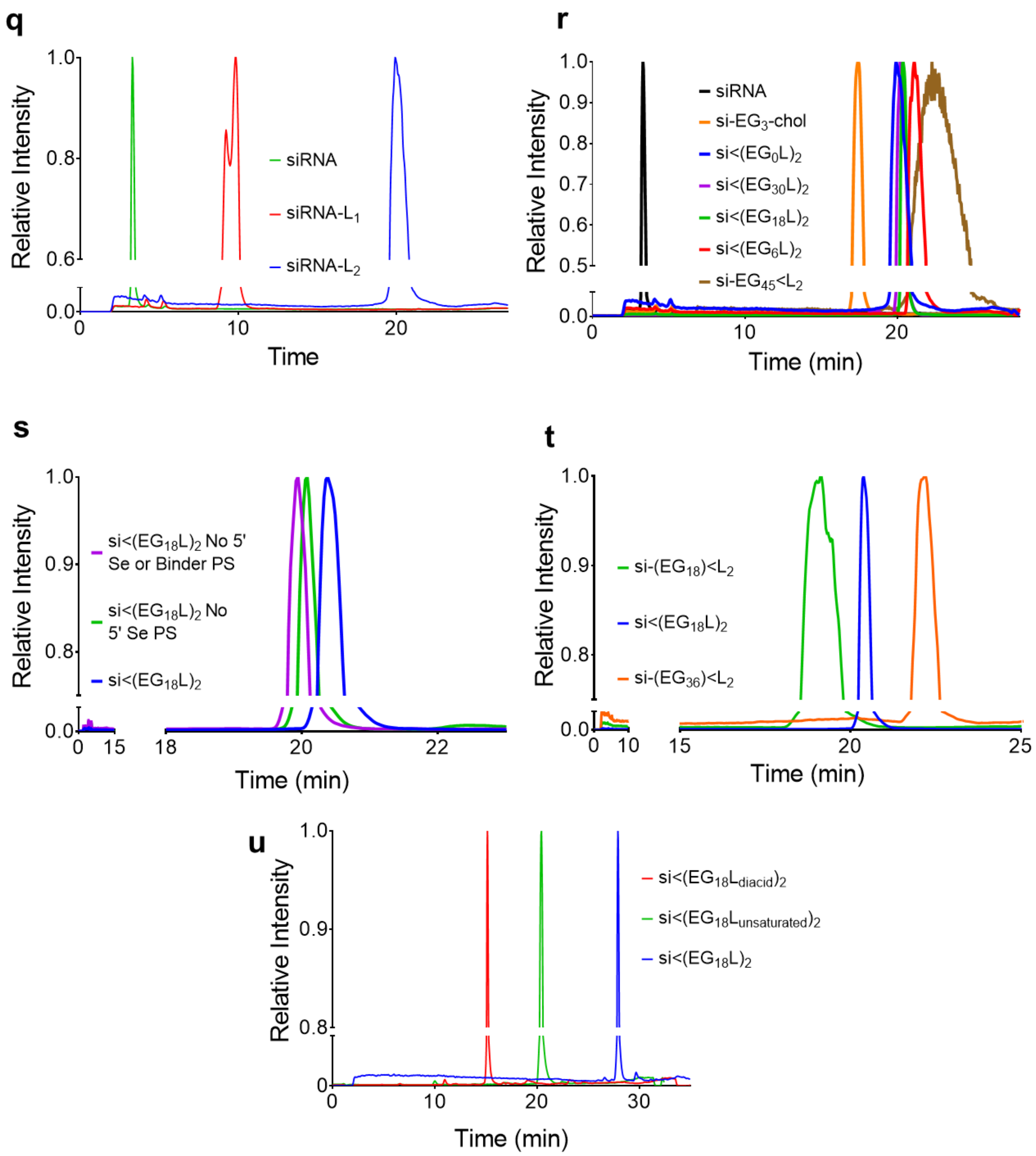

Supplementary Figure 1: LC-MS and HPLC characterization of siRNA and siRNA conjugates. Conjugate purity and mass fidelity were confirmed by LCMS ESI^{-}. LC was performed using a Waters XBridge Oligonucleotide BEH C18 Column under a linear gradient from 85% A (16.3 mM triethylamine – 400 mM hexafluoroisopropanol) to 100% B (methanol) at 45°. (A-P) Full spectrum presented on left and deconvoluted mass presented on right as calculated by ThermoFisher FreeStyle Software. Molecular weight of si<$PEG_{45}L_2$ was validated using MALDI-TOF mass spectrometry. HLPC chromatograms for siRNAs with variations in valency (Q), number of ethylene glycol linker repeats (R), phosporothioate content (S), branching architecture (T), and lipid chemistry (U). Axes are adjusted to enable visualization of differences in elution time.

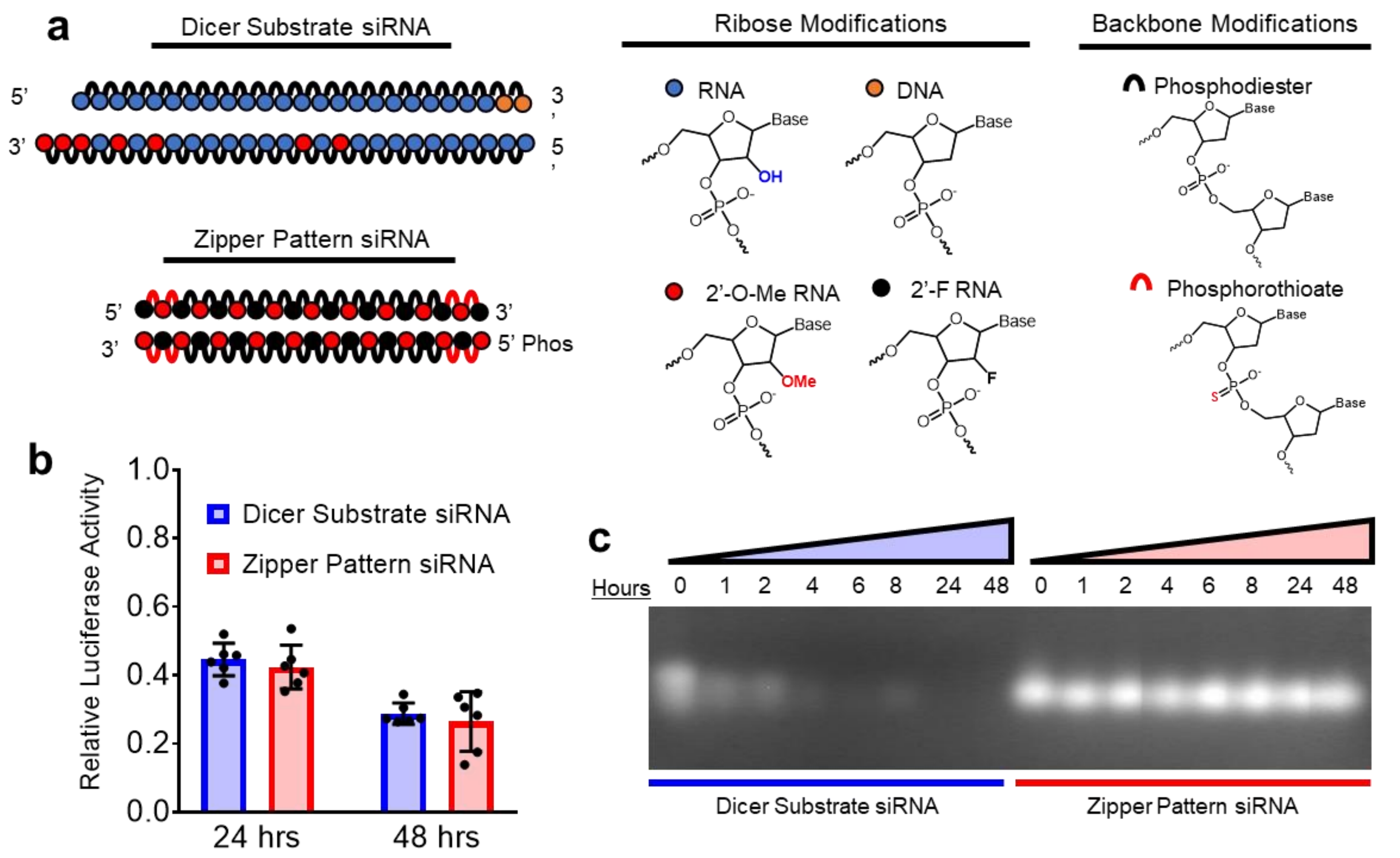

Supplementary Figure 2: **siRNA modification pattern validation and stability.** (A) Graphical schematic of structural differences between synthetic Dicer Substrate siRNA and alternating 2'F-, 2'OMe-modified "Zipper" siRNA used throughout this study. (B) Lipofection-mediated knockdown of 25 nM siRNA in Luciferase-expressing MDA-MB-231s. Luminescent signal was normalized to negative control siRNAs to account for any nonspecific toxicity effects (n=6). (C) Serum stability of siRNA challenged with 60% fetal bovine serum at 37°C visualized by agarose gel electrophoresis.

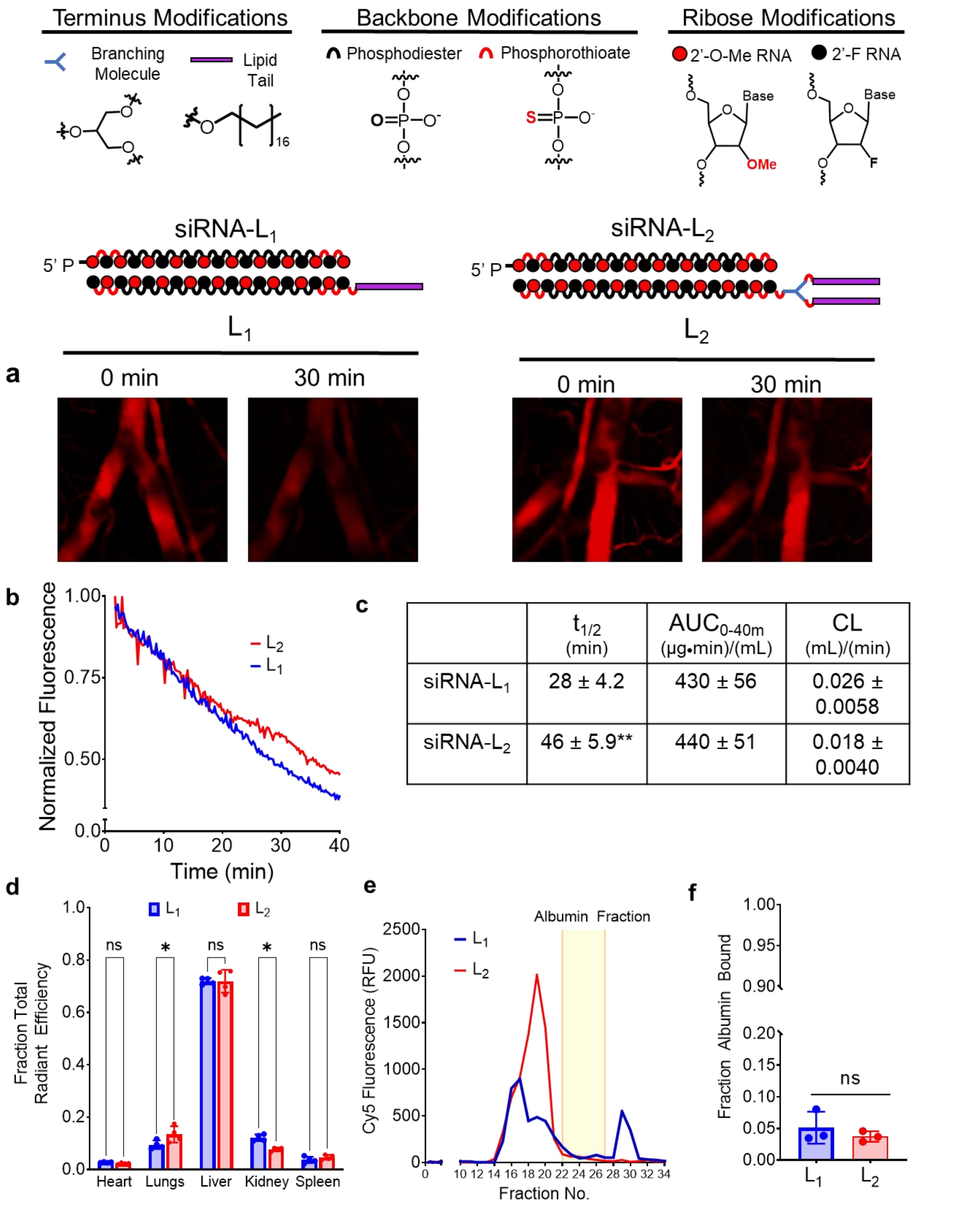

Supplementary Figure 3: Divalent lipid-siRNA conjugate exhibits improved bioavailability compared to monovalent conjugate A) Representative intravital microscopy images of mouse ear vasculature monitored for fluorescence decay after i.v. injection of siRNA conjugate. B) Average of (n=4) intravital fluorescent signals from individual mice displayed. C) Pharmacokinetic parameters of siRNA conjugates determined by intravital

microscopy (D) Biodistribution of fluorescent conjugates approximately 45 minutes after 1 mg/kg intravenous injection as measured by IVIS (n=4). Significance assessed by 2-way ANOVA with Sidak's multiple comparisons tests (E) Plasma samples collected from mice approximately 1h after treatment (1 mg/kg) were fractionated and quantitated for Cy5 fluorescence by size exclusion chromatography. A representative trace is shown of fluorescence signal within each plasma fraction for each siRNA structure; the albumin-containing plasma fractions are highlighted. F) Cy5 fluorescence within the albumin-containing fractions was quantitated relative to total plasma fluorescence fraction of conjugate bound to albumin in mouse plasma after 1 mg/kg intravenous injection as calculated from known standards and sum of fractions' fluorescent intensities (n=3). Significance assessed using Welch's t-test.

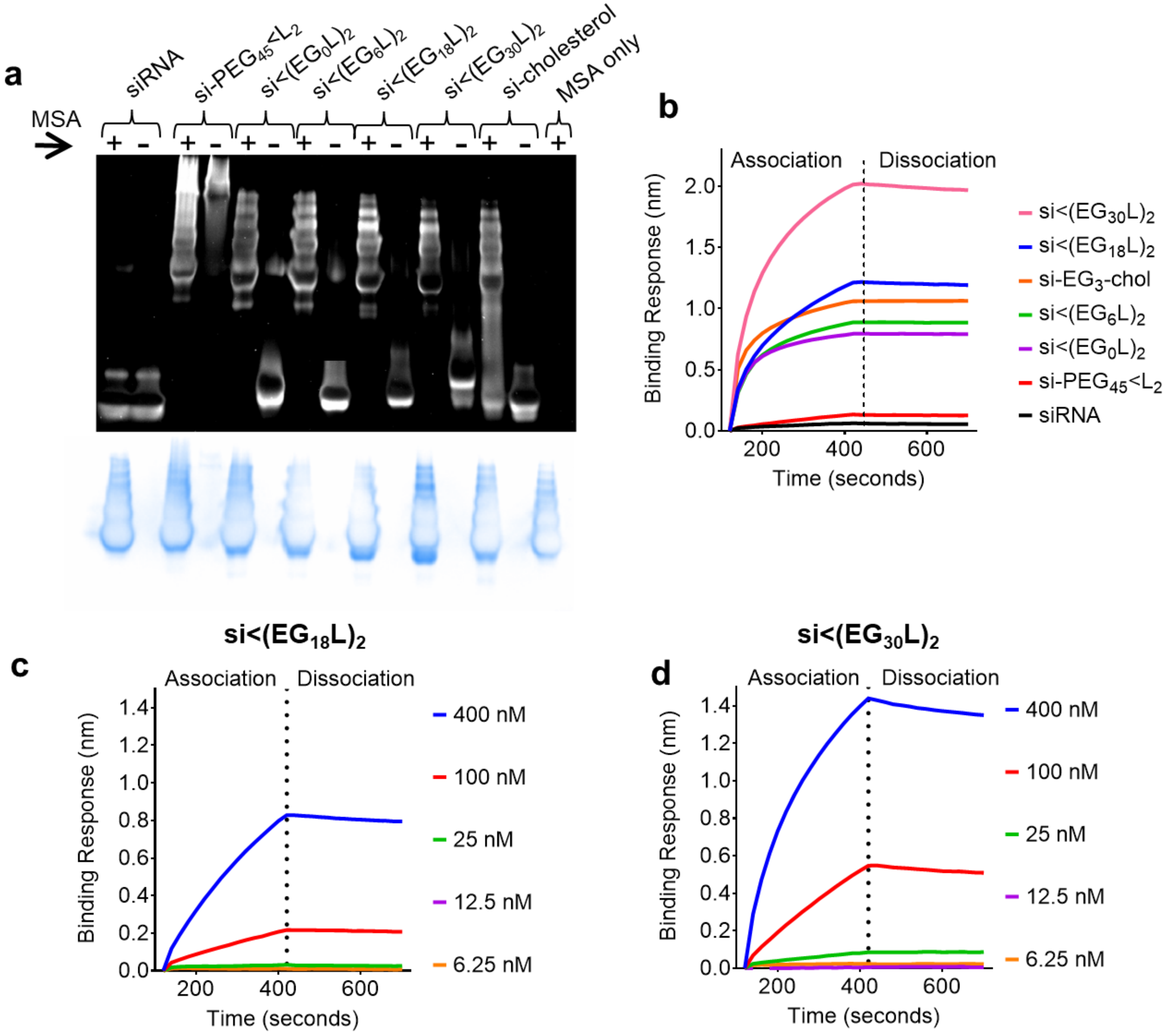

e

	KD (nM)	k_{on} (1/Ms)	k_{off} (1/s)
**si<(EG$_{18}$L)$_2$**	30 ± 0.3	6.97×10^3 ±152	$2.09 \times 10^{-4} \pm 1.75 \times 10^{-6}$
**si<(EG$_{30}$L)$_2$**	9.49 ± 0.1	1.85×10^4 ± 340	$1.75 \times 10^{-4} \pm 2.09 \times 10^{-6}$

Supplementary Figure 4: (A) Native PAGE Gel of siRNA conjugates run in the presence or absence of mouse serum albumin (MSA). Albumin colocalization can be seen by an upwards shift of nucleic acid staining

to coincide with albumin (indicated in bottom panel by staining of the same gel with Coomassie blue). (B) Binding response of siRNA conjugates to 400 nM mouse serum albumin as measured by biolayer interferometry. Full panel of (C) si<(EG$_{18}$L)$_2$ and (D) si<(EG$_{36}$L)$_2$ binding responses to HSA used to determine (E) binding kinetic parameters.

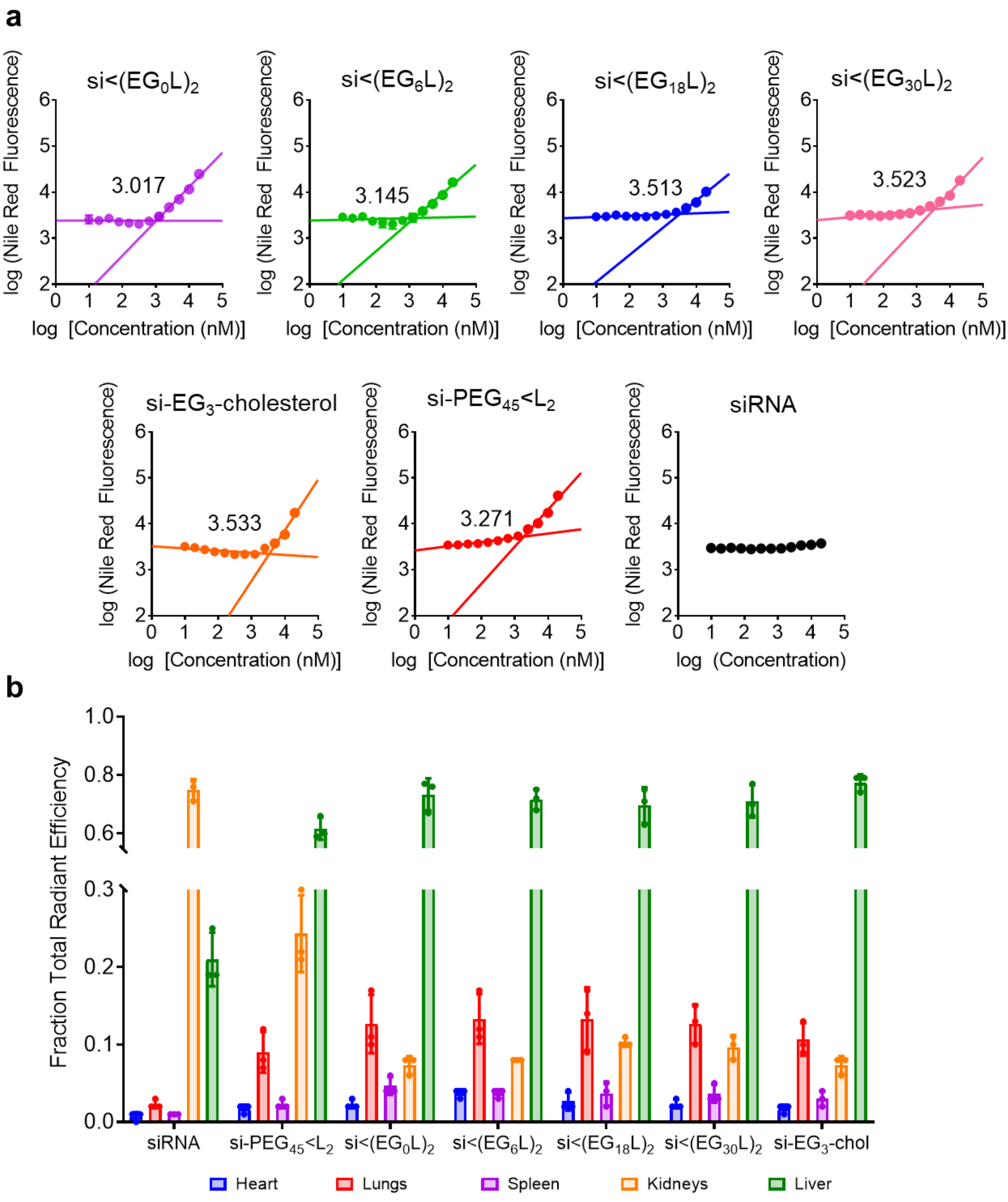

Supplementary Figure 5: (A) Critical micelle concentration (CMC) plots for siRNA conjugate library. (B) Biodistribution of Cy5-labeled siRNA conjugates in non-tumor bearing mice. Organs harvested approximately 1 h after injection at 1 mg/kg intravenously *via* tail vein (n=3).

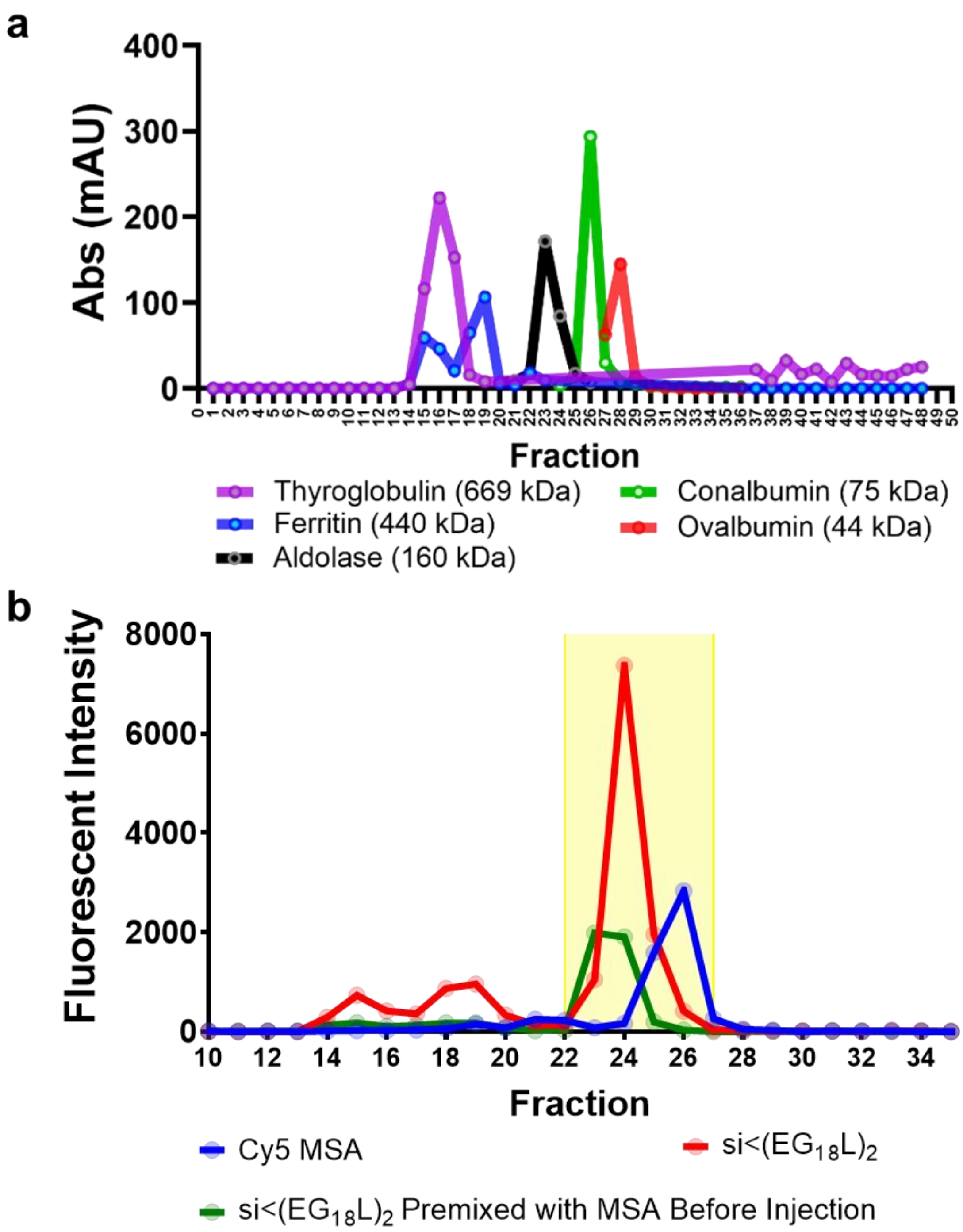

Supplementary Figure 6: (A) Size exclusion chromatography (SEC) of protein standards. Representative protein standards run on SEC system to demonstrate resolution by molecular weight. (B) Size exclusion chromatography (SEC) of mouse plasma after injection with Cy5-siRNA conjugate +/- native MSA versus Cy5-MSA alone. Mice were injected at 1 mg/kg intravenously via tail vein and plasma was harvested approximately 1h after injection**.** Fractions highlighted in yellow were used for calculating albumin-bound fraction.

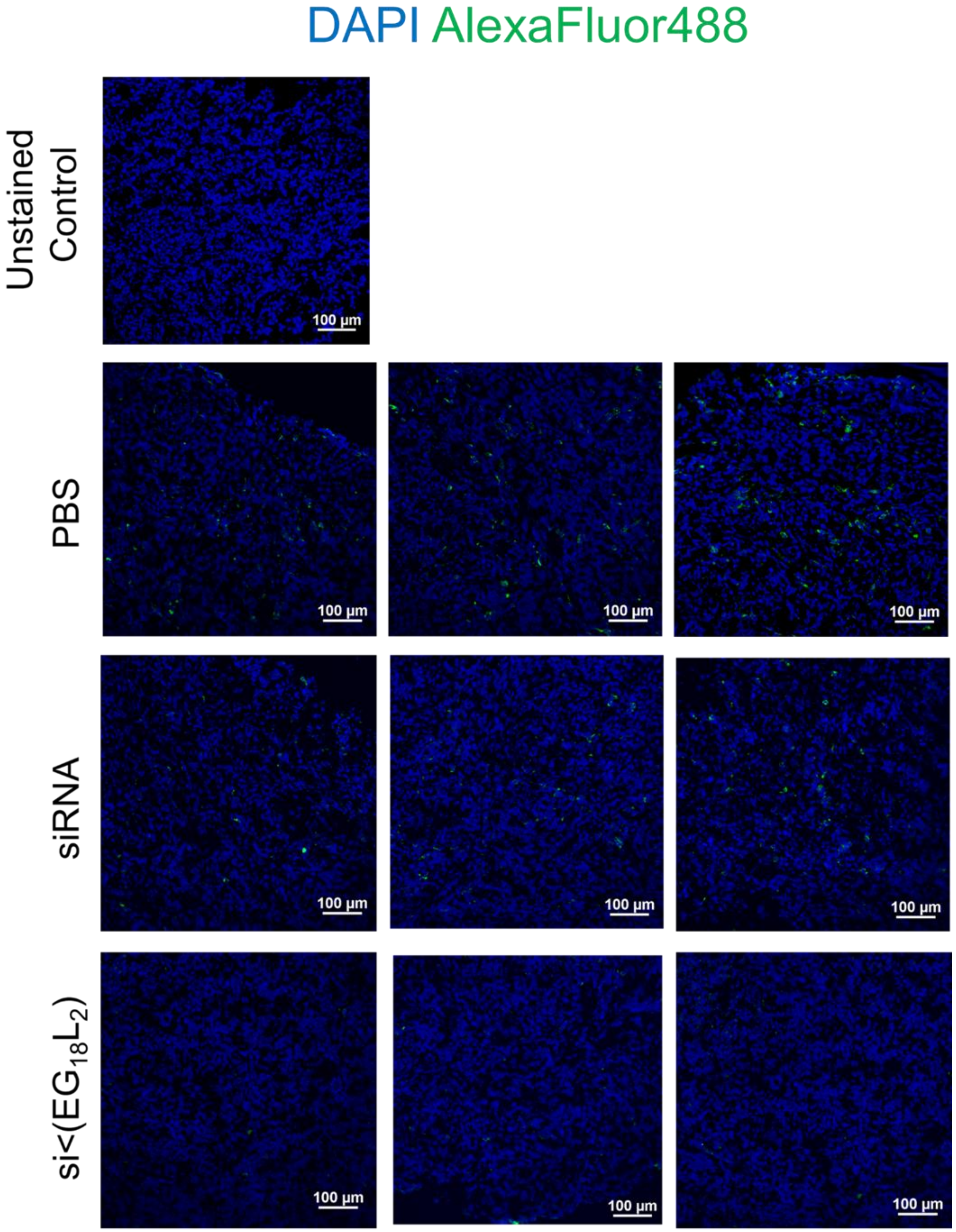

Supplementary Figure 7: Extended images of tumors sectioned and immunohistochemically stained for Firefly Luciferase protein (green, Alexafluor488) and cell nuclei (blue, DAPI).

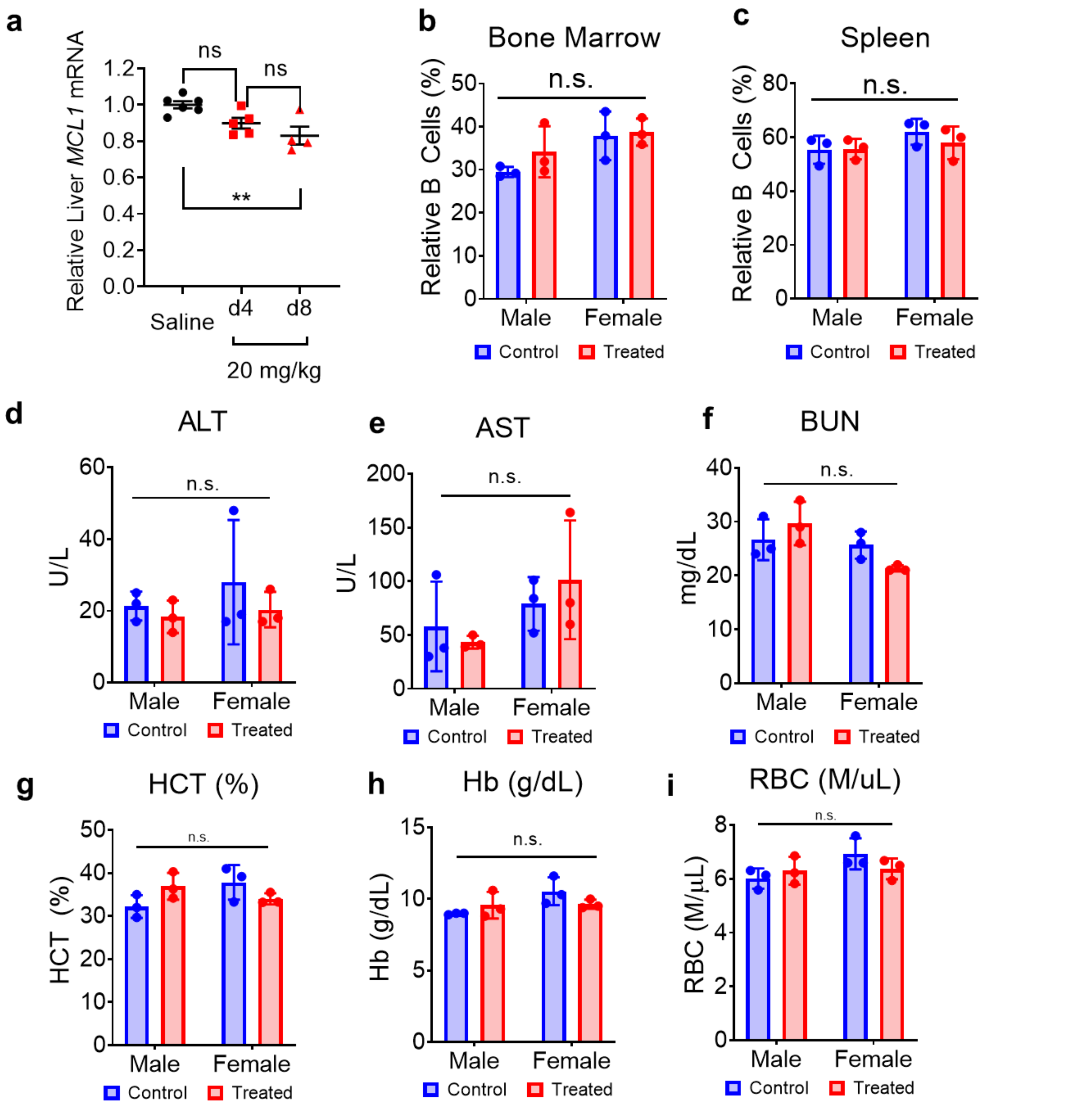

Supplementary Figure 8: (A) Liver knockdown of Mcl-1 mRNA after bolus i.v. injection of si_{Mcl-1}<$(EG_{18}L)_2$. Significance assessed by 1-way ANOVA with Tukey's multiple comparisons test (n=4-6). B cells relative to total viable cells isolated from (B) bone marrow and (C) spleen isolated from wild type mice three days after injection with 20 mg/kg siRNA conjugate targeting Mcl-1 compared to untreated controls. Levels of blood chemistry markers of toxicity including (D) alanine aminotransferase, (E) aspartate aminotransferase, and (F) blood urea nitrogen were measured on sera of treated and untreated mice. Significance assessed by 2-way ANOVA with Tukey's multiple comparisons test (n=3). (G-I) Complete blood count values collected from mice 3 days after injection with 20 mg/kg of siRNA conjugate targeting Mcl-1. Significance assessed by 2-way ANOVA (n=3).

a

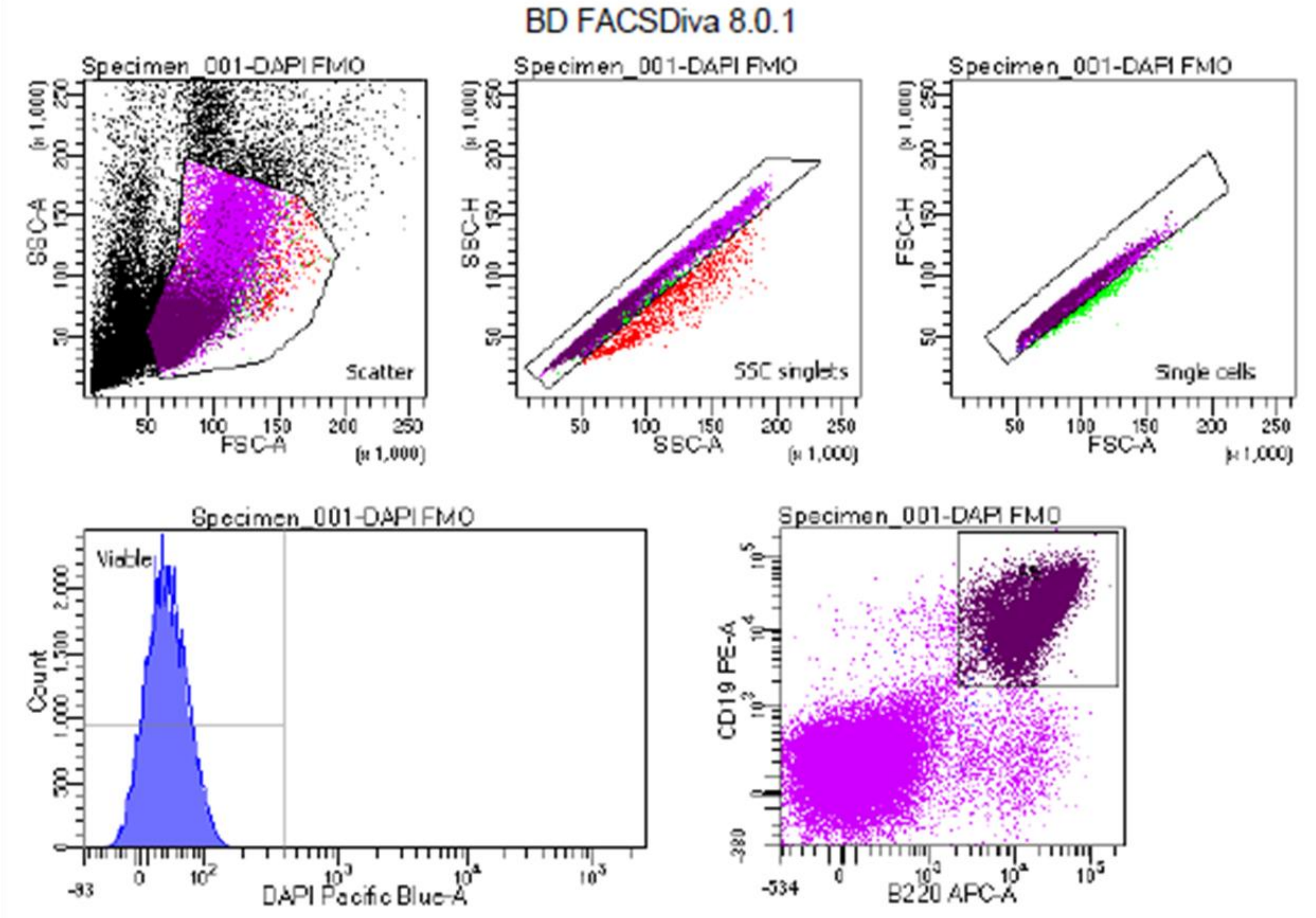

Tube: DAPI FMO

Population	#Events	%Parent	%Total
All Events	81,448	####	100.0
Scatter	52,307	64.2	64.2
SSC singlets	51,112	97.7	62.8
Single cells	50,376	98.6	61.9
Viable	50,302	99.9	61.8
P5	23,910	47.5	29.4

b

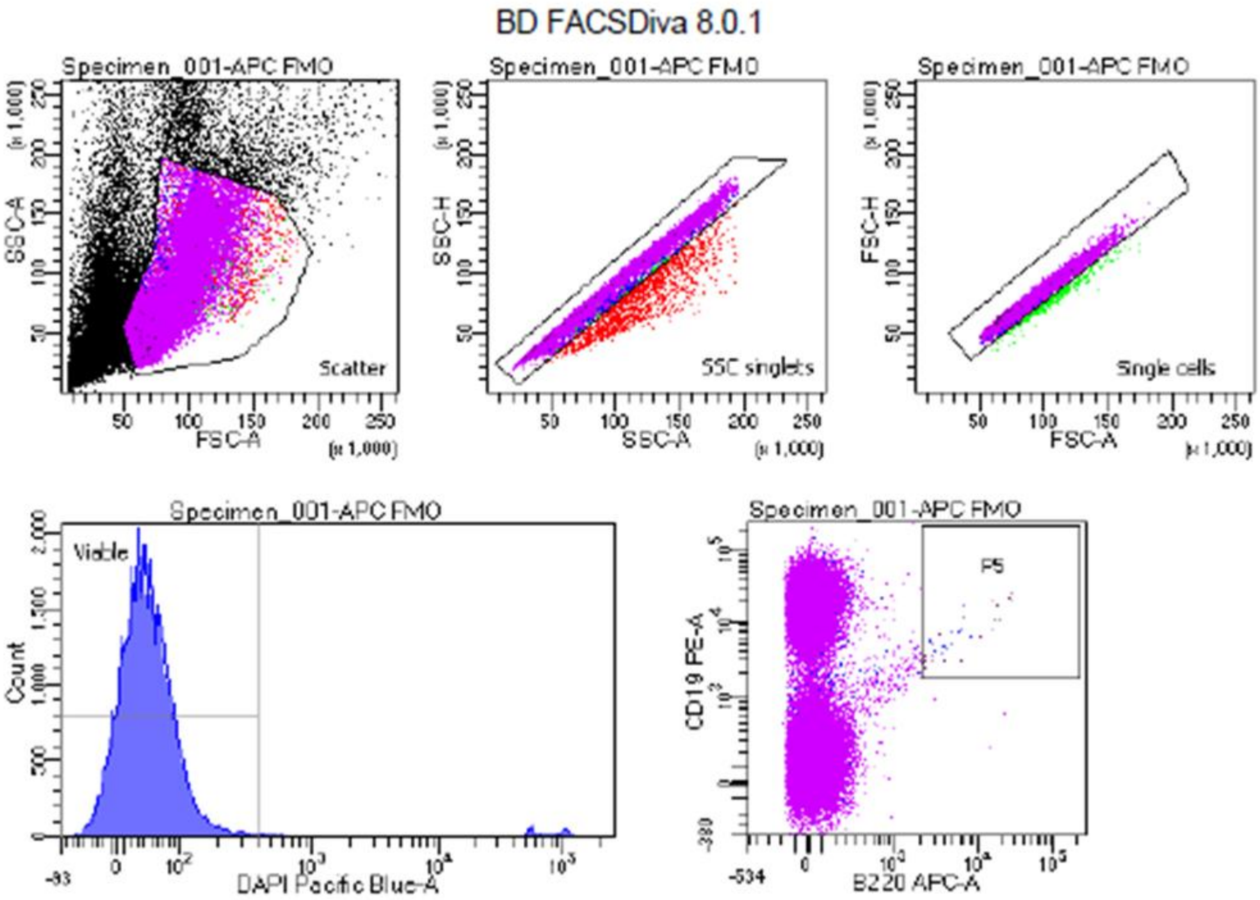

Tube: APC FMO

Population	#Events	%Parent	%Total
All Events	91,421	####	100.0
Scatter	54,222	59.3	59.3
SSC singlets	52,697	97.2	57.6
Single cells	52,084	98.8	57.0
Viable	50,115	96.2	54.8
P5	26	0.1	0.0

c

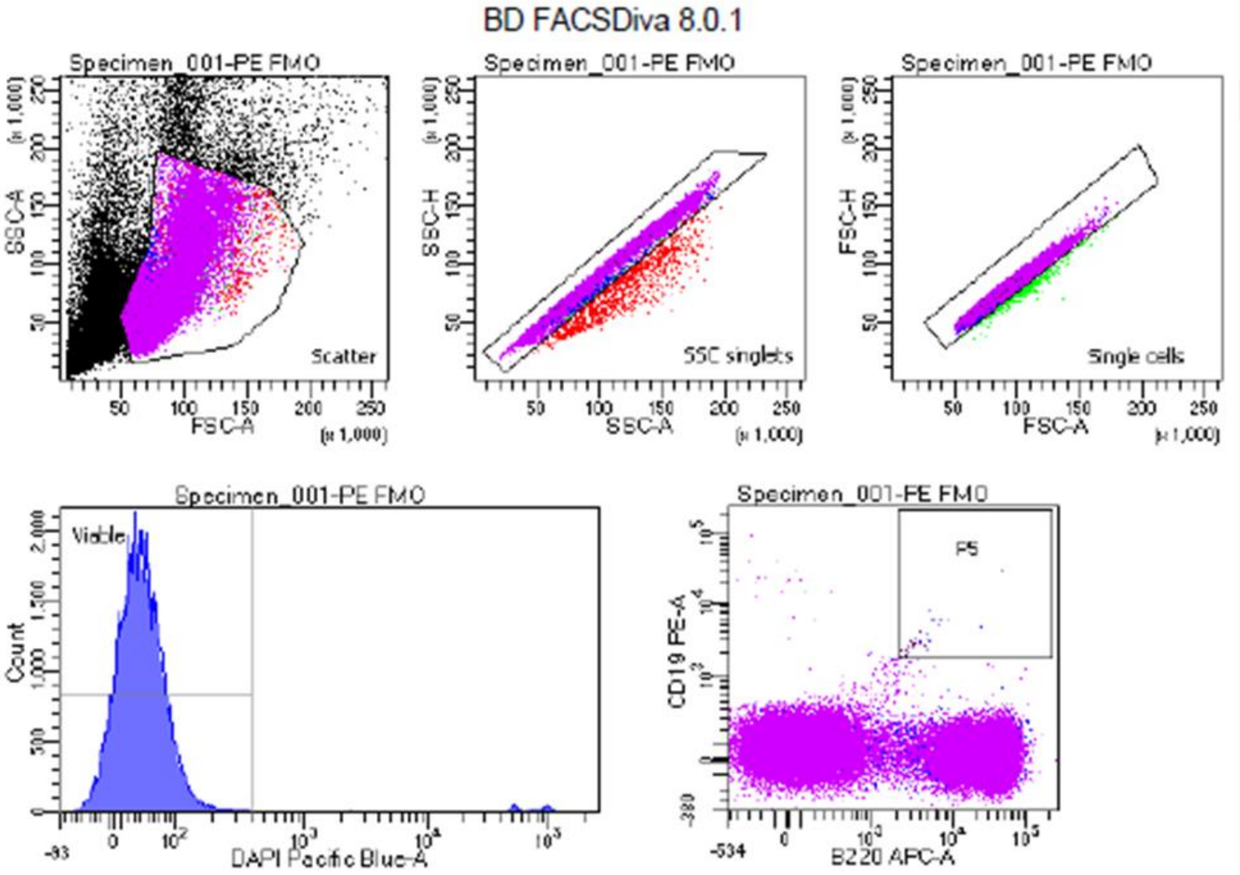

Tube: PE FMO

Population	#Events	%Parent	%Total
All Events	87,769	####	100.0
Scatter	53,736	61.2	61.2
SSC singlets	52,424	97.6	59.7
Single cells	51,826	98.9	59.0
Viable	50,085	96.6	57.1
P5	23	0.0	0.0

d

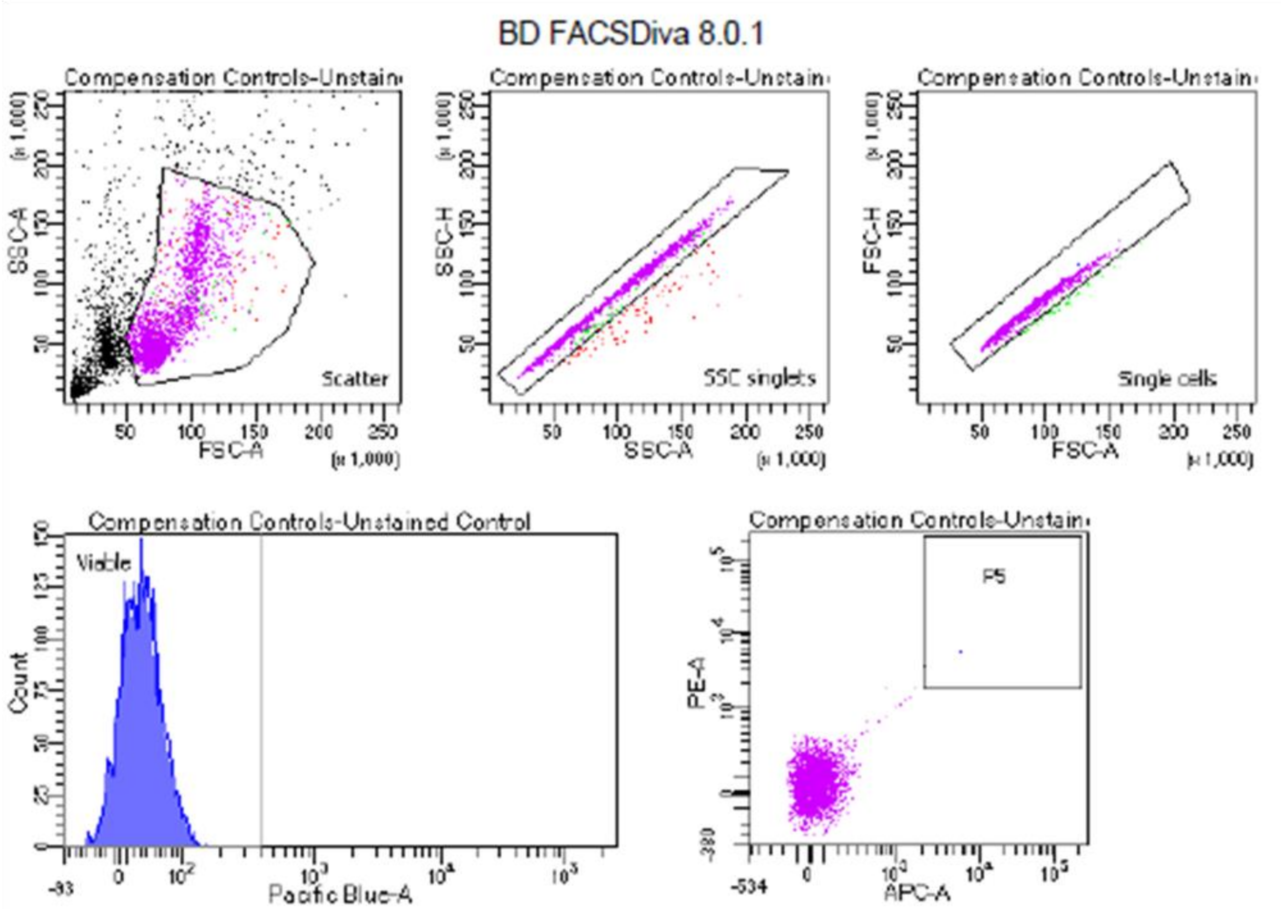

Tube: Unstained Control

Population	#Events	%Parent	%Total
All Events	5,000	####	100.0
Scatter	3,128	62.6	62.6
SSC singlets	3,042	97.3	60.8
Single cells	2,992	98.4	59.8
Viable	2,991	100.0	59.8
P5	1	0.0	0.0

e

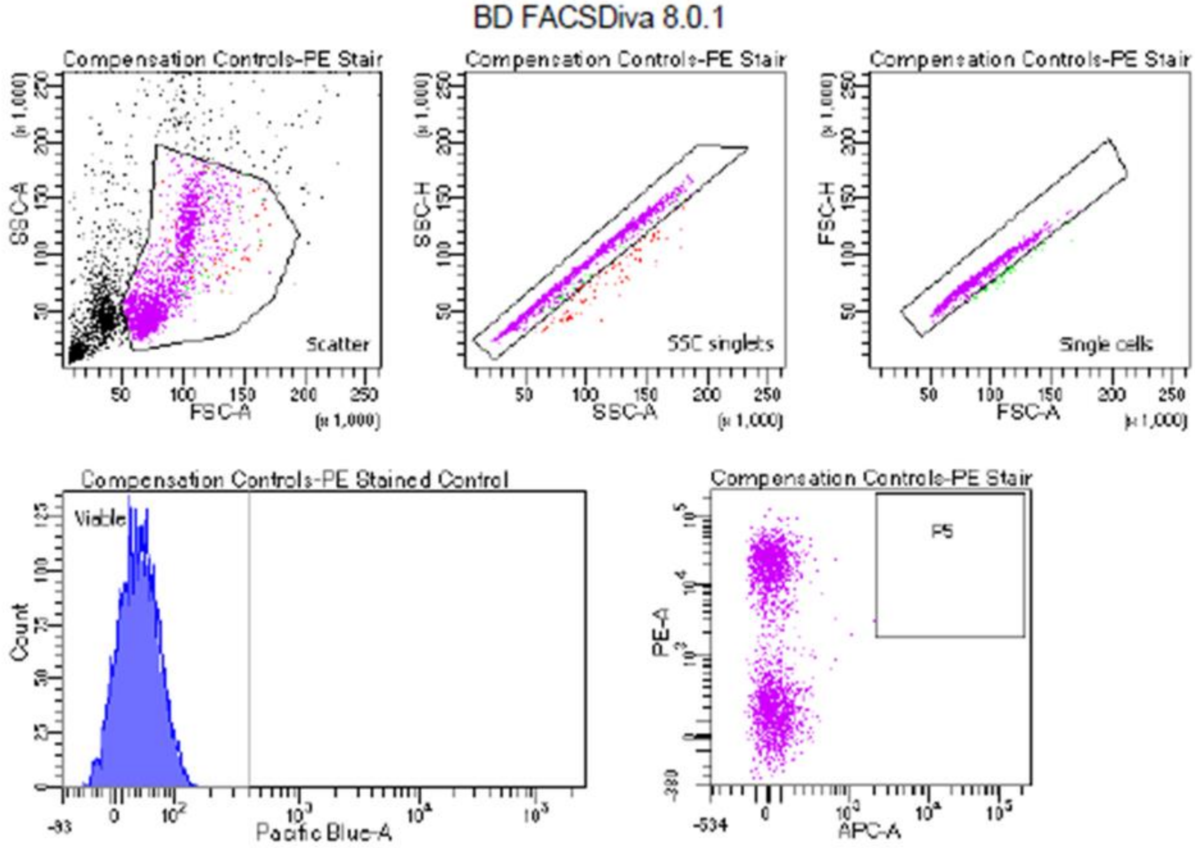

Tube: PE Stained Control

Population	#Events	%Parent	%Total
All Events	5,000	####	100.0
Scatter	3,075	61.5	61.5
SSC singlets	2,974	96.7	59.5
Single cells	2,925	98.4	58.5
Viable	2,925	100.0	58.5
P5	0	0.0	0.0

f

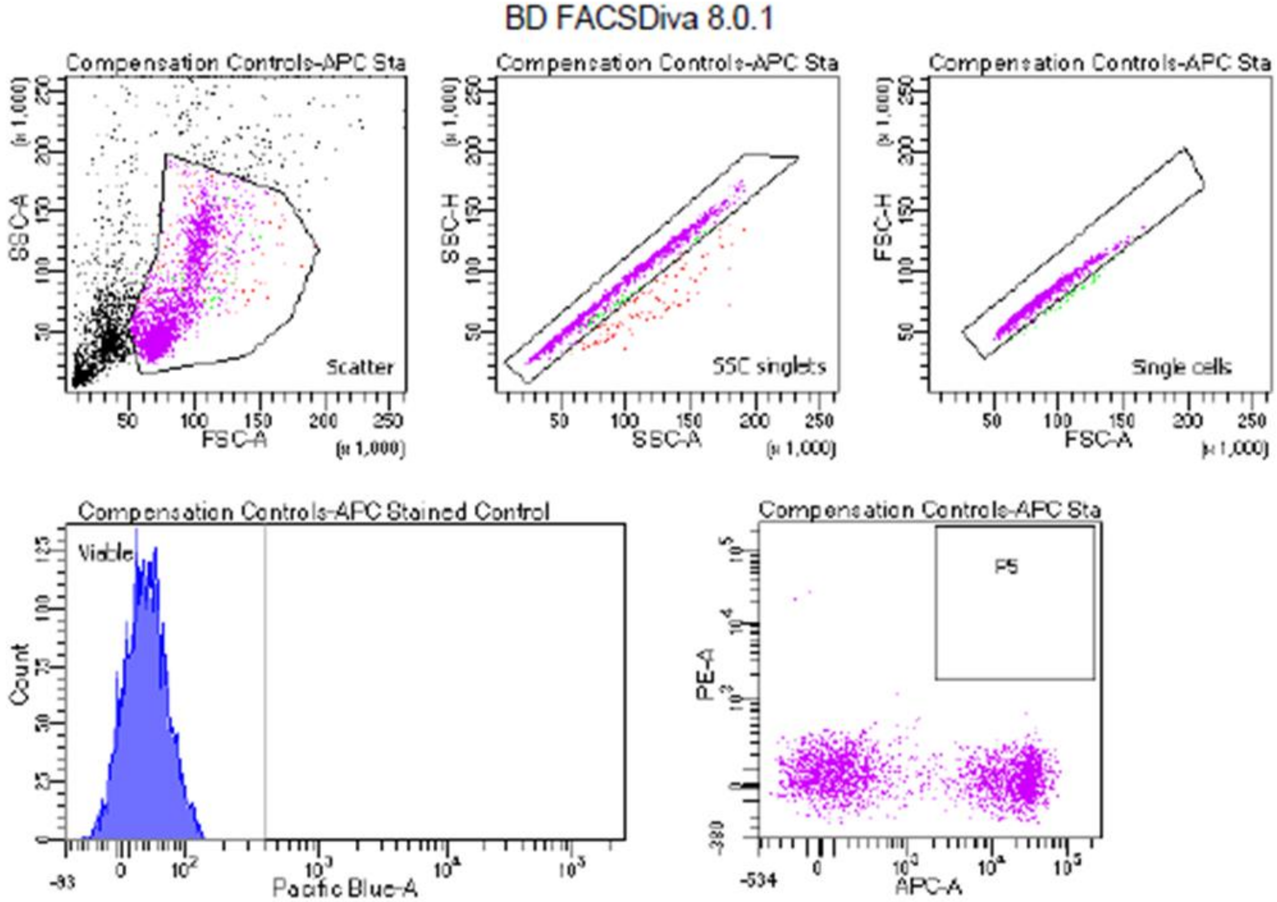

Tube: APC Stained Control

Population	#Events	%Parent	%Total
All Events	5,000	####	100.0
Scatter	3,005	60.1	60.1
SSC singlets	2,907	96.7	58.1
Single cells	2,862	98.5	57.2
Viable	2,862	100.0	57.2
P5	0	0.0	0.0

g

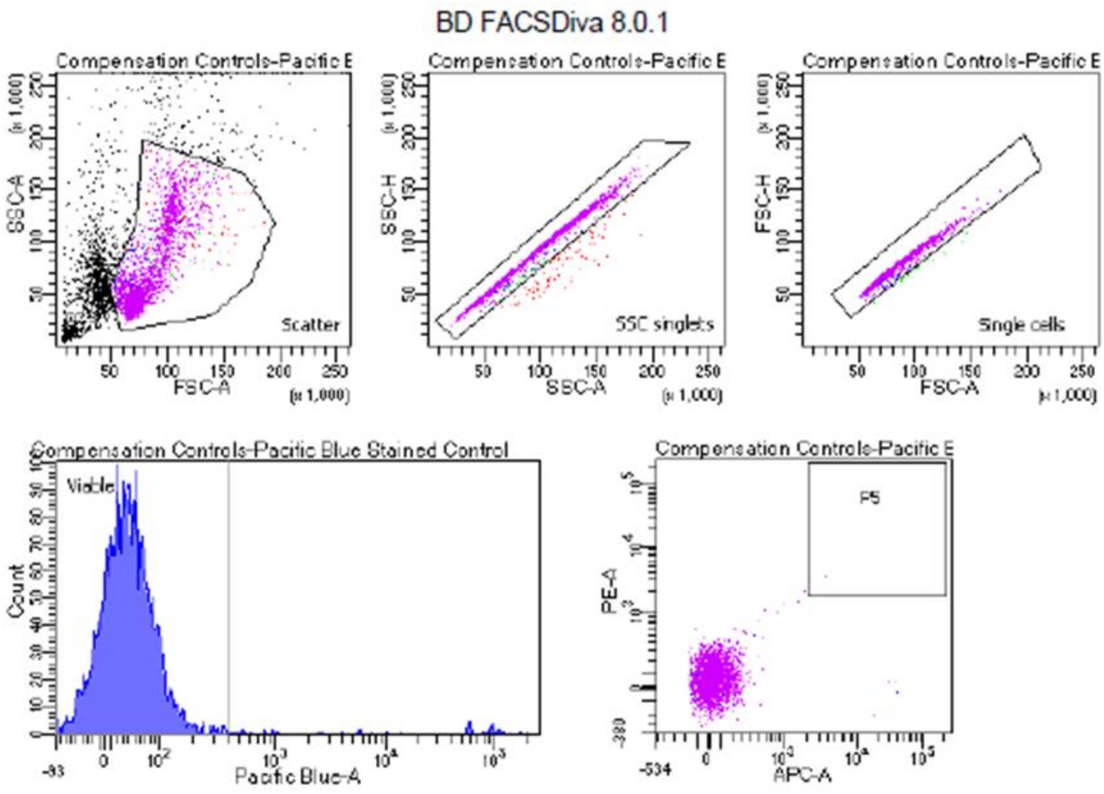

Tube: Pacific Blue Stained Control

Population	#Events	%Parent	%Total
All Events	5,000	####	100.0
Scatter	2,942	58.8	58.8
SSC singlets	2,861	97.2	57.2
Single cells	2,829	98.9	56.6
Viable	2,730	96.5	54.6
P5	1	0.0	0.0

Supplementary Figure 9: Flow gating for B cells harvested from mouse spleens and livers. Fluorescence Minus One (FMO) controls using pooled cells from all samples for (A) DAPI, (B) APC, and (C) PE antibodies to set boundaries for background signal. (D) Unstained pooled cell control. (E-G) PE, APC, and DAPI alone stained pooled cell controls used to inform gating.

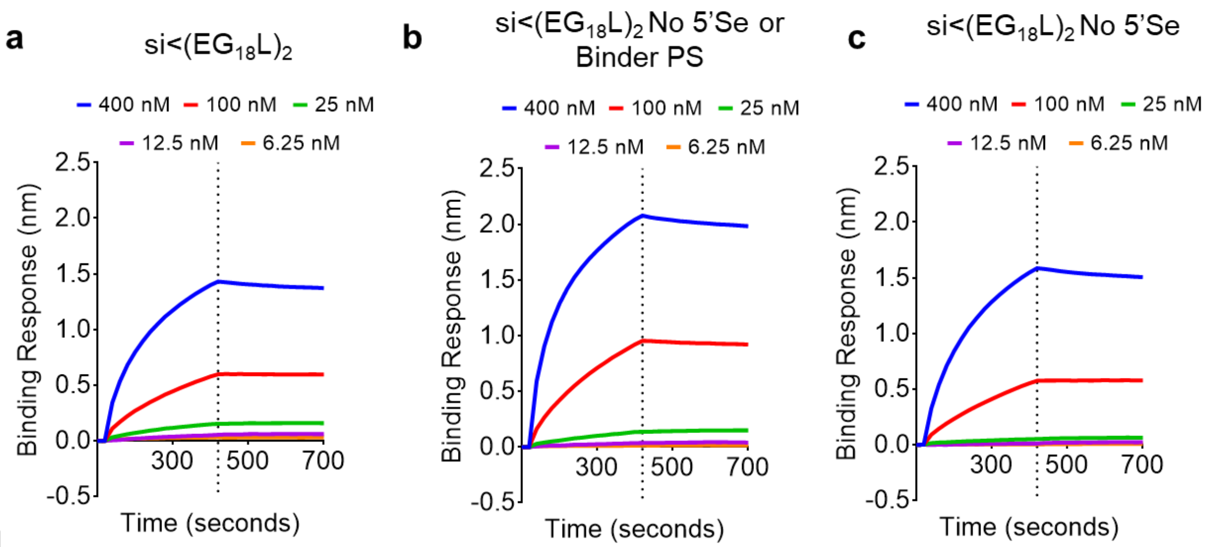

d

	KD (nM)	k_{on} (1/Ms)	k_{off} (1/s)
**si<($EG_{18}L)_2$**	3.89 ± 0.13	$2.35x10^4$ ± 6.37	$9.14x10^{-5}$ ± $3.09x10^{-6}$
**si<($EG_{18}L)_2$ No 5'Se or Binder PS**	2.81 ± 0.10	$3.01x10^4$ ± 7.47	$8.46x10^{-5}$ ± $3.05x10^{-6}$
**si<($EG_{18}L)_2$ No 5'Se**	5.58 ± 0.10	$2.08x10^4$ ± 3.91	$1.16x10^{-4}$ ± $2.07x10^{-6}$

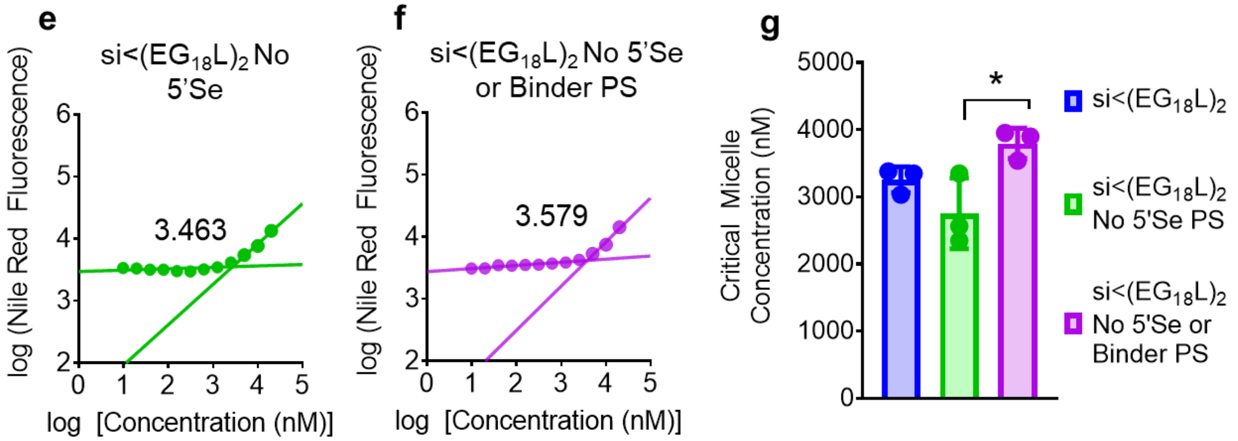

Supplementary Figure 10: Extended characterization of si<($EG_{18}L)_2$ phosphorothioate variants. (A-C) Full panel of binding responses to HSA at varied concentrations measured by biolayer interferometry used to determine (D) binding kinetic parameters. (E-G) Critical micelle concentration observed after 2-hour incubation with Nile Red at 37°C (n=3). Significance assessed by 1-way ANOVA with Tukey's multiple comparisons test.

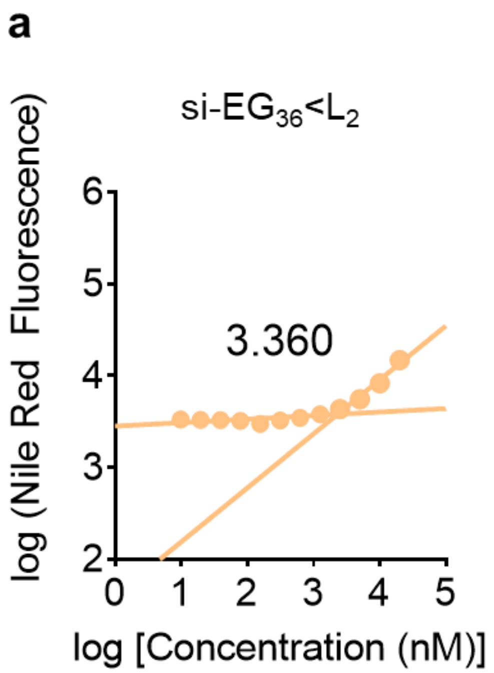

Supplementary Figure 11: Critical micelle concentration (CMC) plots for (A) si-EG_{36}<L_2 and (B) si-EG_{18}<L_2

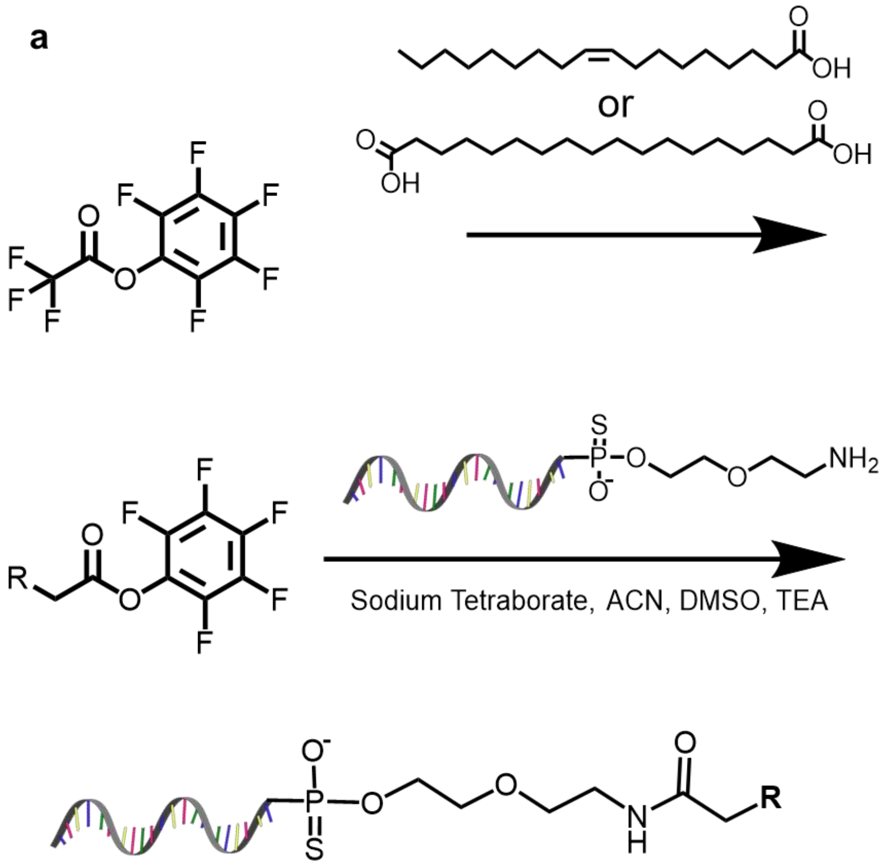

b

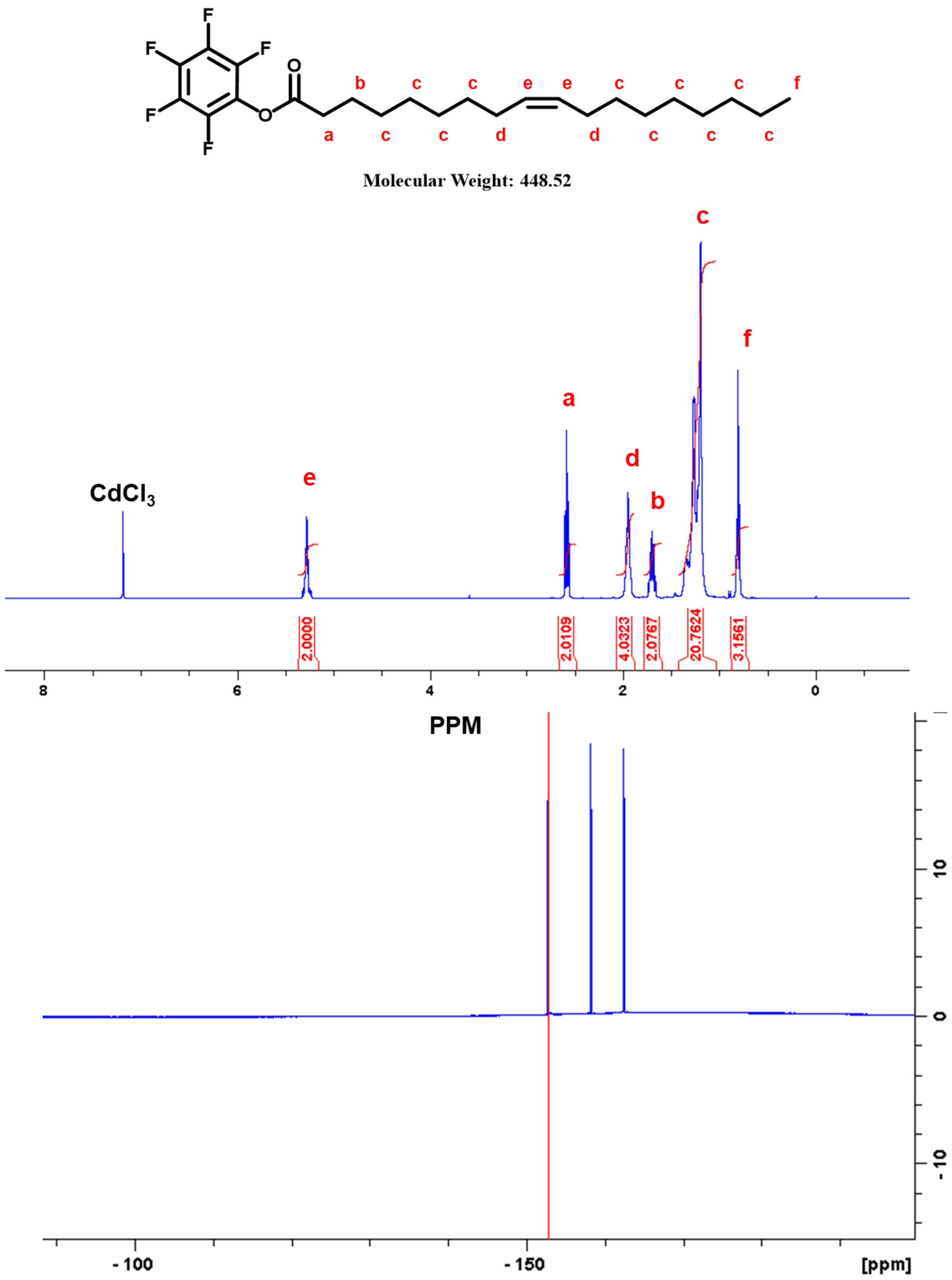
F
F
F
F
F
O
O
a
b
c
d
e
f
Molecular Weight: 448.52
CdCl3
2.0000
2.0109
4.0323
2.0767
20.7624
3.1561
8
6
4
2
0
PPM
10
0
-10
- 100
- 150
[ppm]

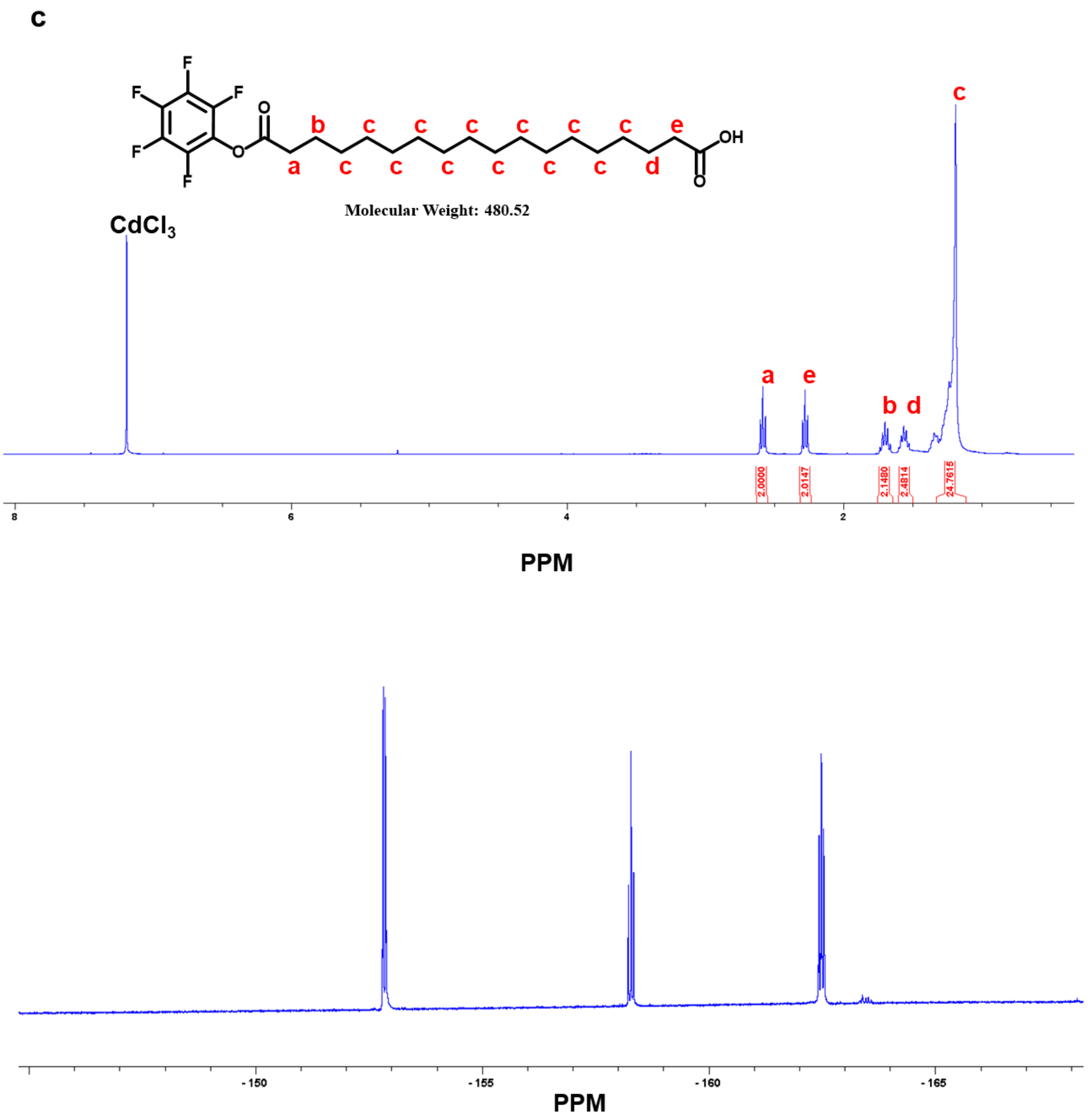

Supplementary Figure 12: Synthesis of si<(EG$_{18}$L)$_2$ lipid variants: (A) Overview of PFPA mediated lipid conjugation with (B) unsaturated lipid and (C) carboxylic acid containing lipid. For each lipid, characterization is shown for ^{1}H NMR (top panel) and ^{19}F NMR (bottom panel).

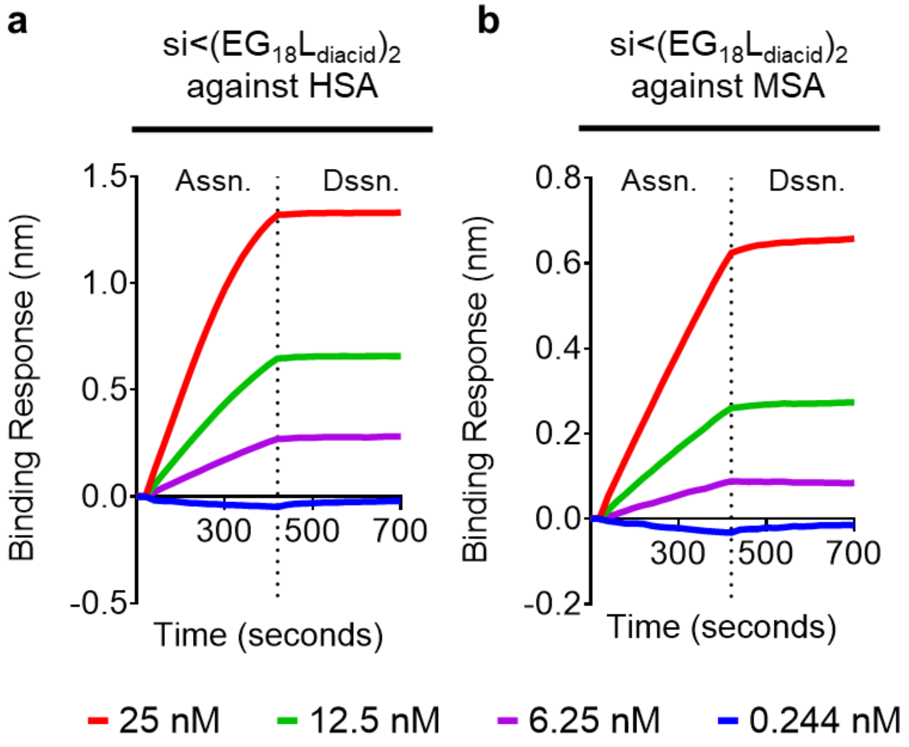

c

HSA Binding Kinetics

	$si<(EG_{18}L_{diacid})_2$	$si<(EG_{18}L)_2$
**KD (nM)**	0.15 ± 0.002	30 ± 0.3
**ka (1/Ms)**	1.31×10^5 ± 712	6.97×10^3 ±152
**kdis (1/s)**	1.93×10^{-5} ± 2.92×10^{-6}	2.09×10^{-4} ± 1.75×10^{-6}

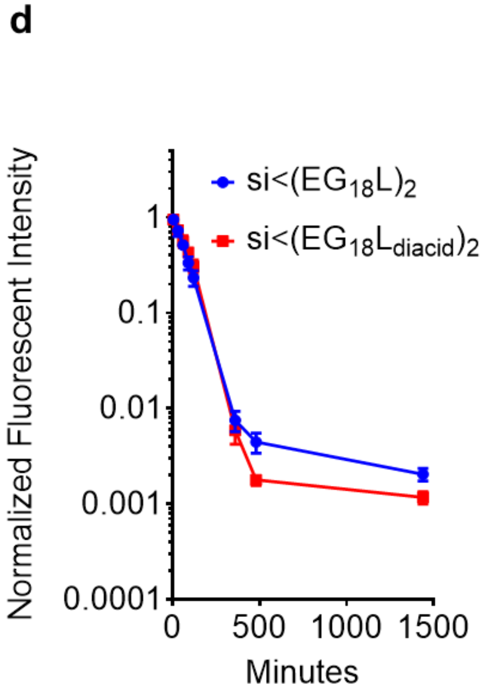

Supplementary Figure 13: Extended characterization of diacid-modified $si<(EG_{18}L)_2$. (A-B) Binding responses to HSA and MSA at varied concentrations measured by biolayer interferometry were used to determine (C) binding kinetic parameters. (D) Mice were injected intravenously with 5 mg/kg of Cy5-labeled siRNA conjugate (n=3-4). At various timepoints, blood was sampled from the contralateral tail vein from injection. Fluorescence was measured from blood samples to determine extended time course PK profiles.

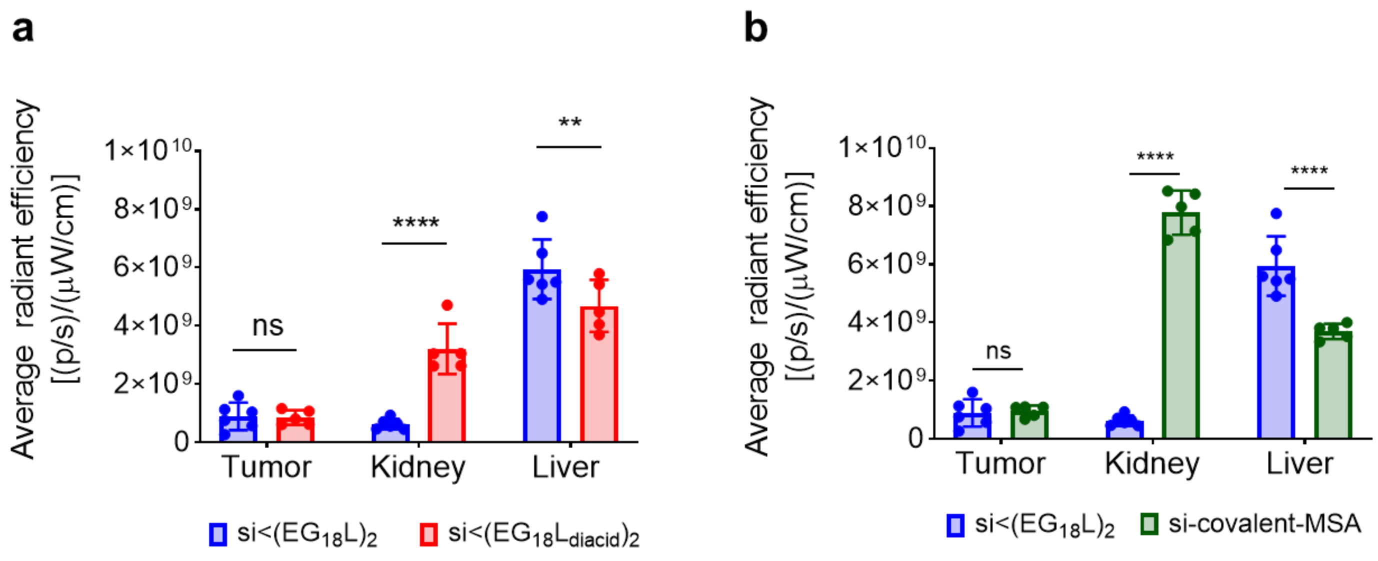

Supplementary Figure 14: Biodistribution of diacid and covalent siRNA conjugates in tumor bearing mice. Epifluorescence of whole organs measured by IVIS comparing accumulation of fluorescently labeled (A) si<(EG$_{18}$L$_{diacid}$)$_2$ and (B) si-covalent-MSA to lead conjugate si<(EG$_{18}$L)$_2$ 18h after i.v. injection of 1 mg/kg siRNA.

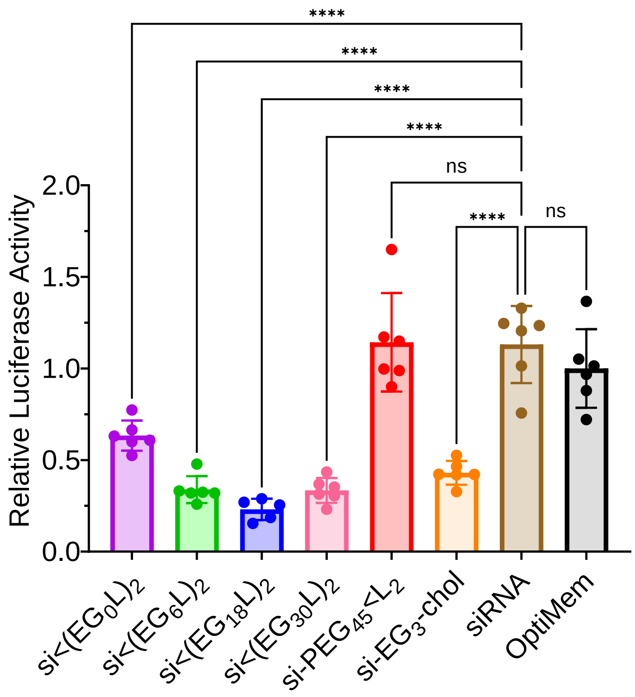

Supplementary Figure 15: Carrier-free knockdown after 72h treatment with 1 μM siRNA in Luciferase-expressing MDA-MB-231s. Luminescent signal was normalized to cells treated with OptiMEM alone. Significance assigned using Ordinary one-way ANOVA with Dunnett's multiple comparisons test using siRNA alone as the group for comparison.

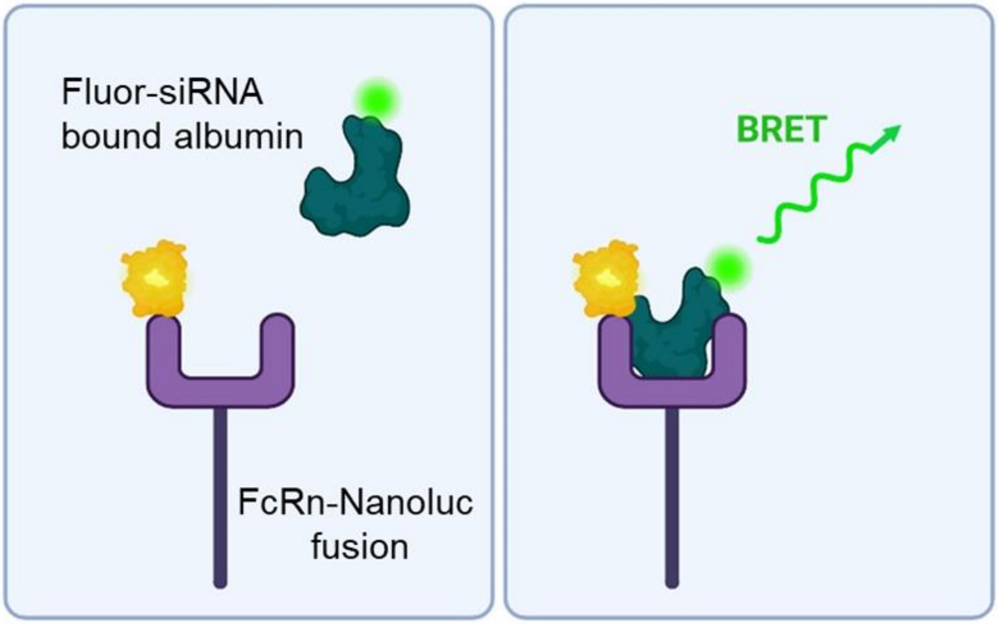

Supplementary Figure 16: Schematic representation of NanBRET assay for assessing interactions of albumin bound Cy5-labeled siRNA<$(EG_{18}L)_2$ with the FcRn.

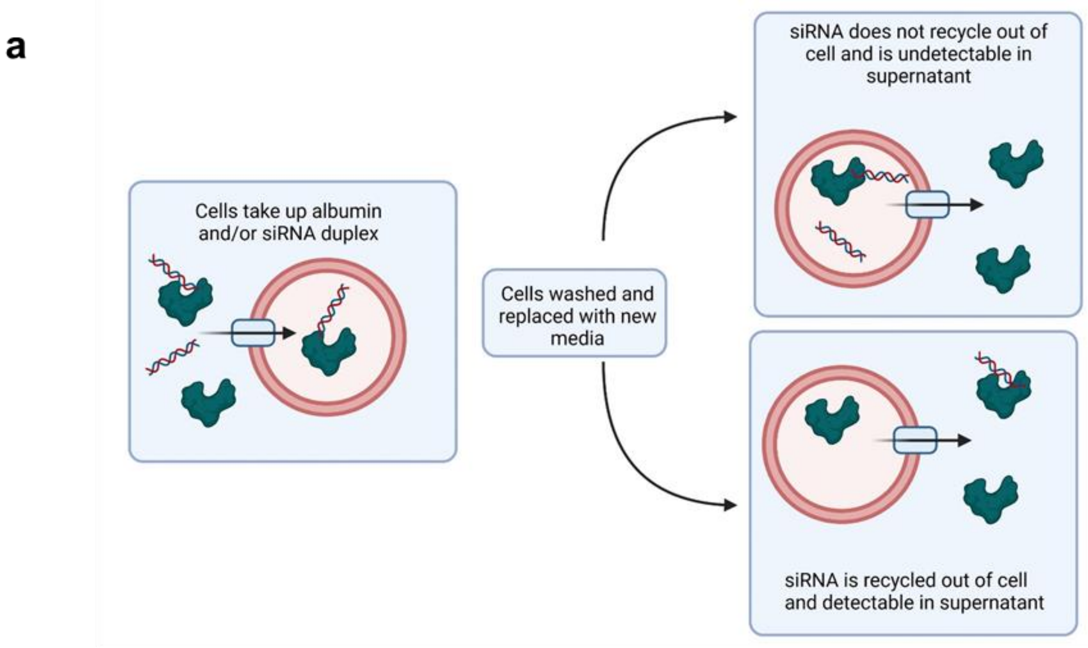

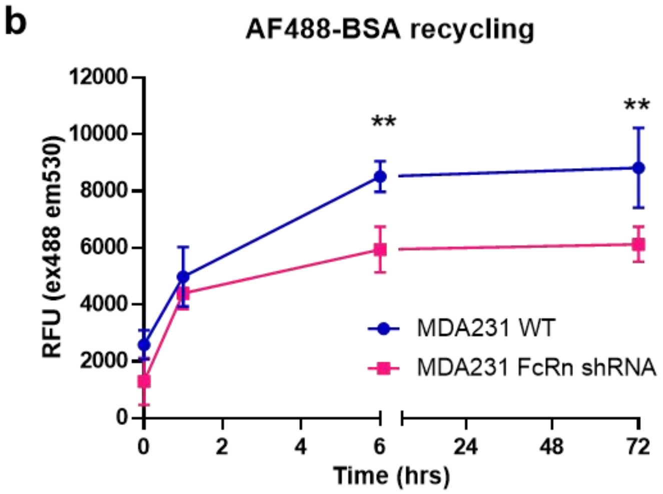

Supplementary Figure 17: (A) Schematic representation of study design for measurement of FcRn mediated extracellular recycling of siRNA<($EG_{18}L)_2$. (B) Recycling of Alexa488-BSA in either WT or FcRn shRNA MDA-MB-231 cells after treatment at 500 nM for 24h. Fluorescence in media supernatant was measured at 0, 1, 6, and 24h.

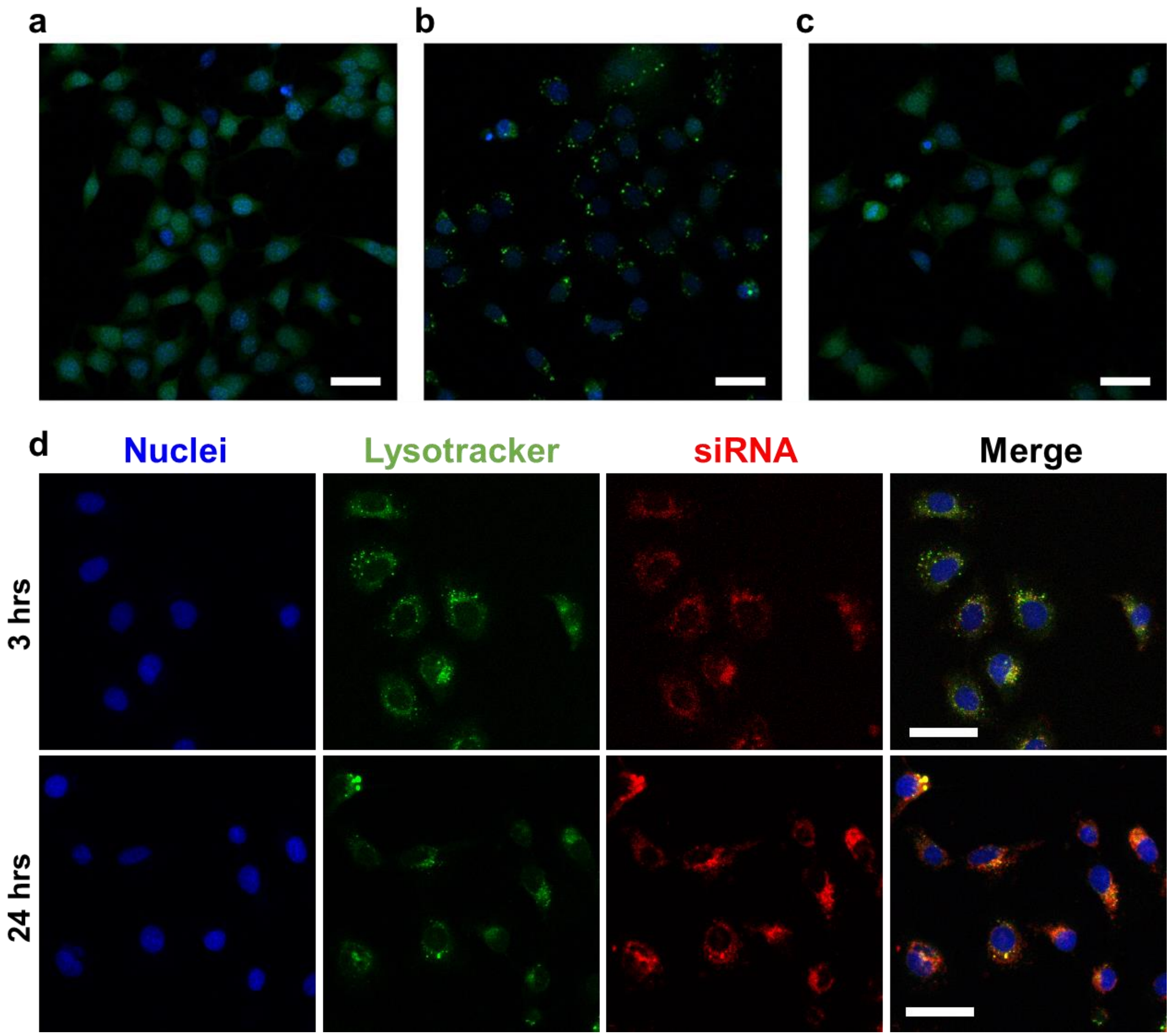

Supplementary Figure 18: Representative images of MDA-MB-231 cells expressing YFP-Gal8 after 18h treatment with (A) media only, (B) lipofectamine (positive control), or (C) si<($EG_{18}L$)$_2$. Gal8 is shown in green, NucBlue nuclear dye in blue. Punctate Gal8 foci indicate endosomal disruption. Scale bar = 50 µm. (D) Representative confocal microscopy images showing qualitative localization of Cy5-labeled si<($EG_{18}L$)$_2$ in MDA-MB-231 cells, relative to endosomes (punctate staining with Lysotracker dye). Scale bar = 50 µm.

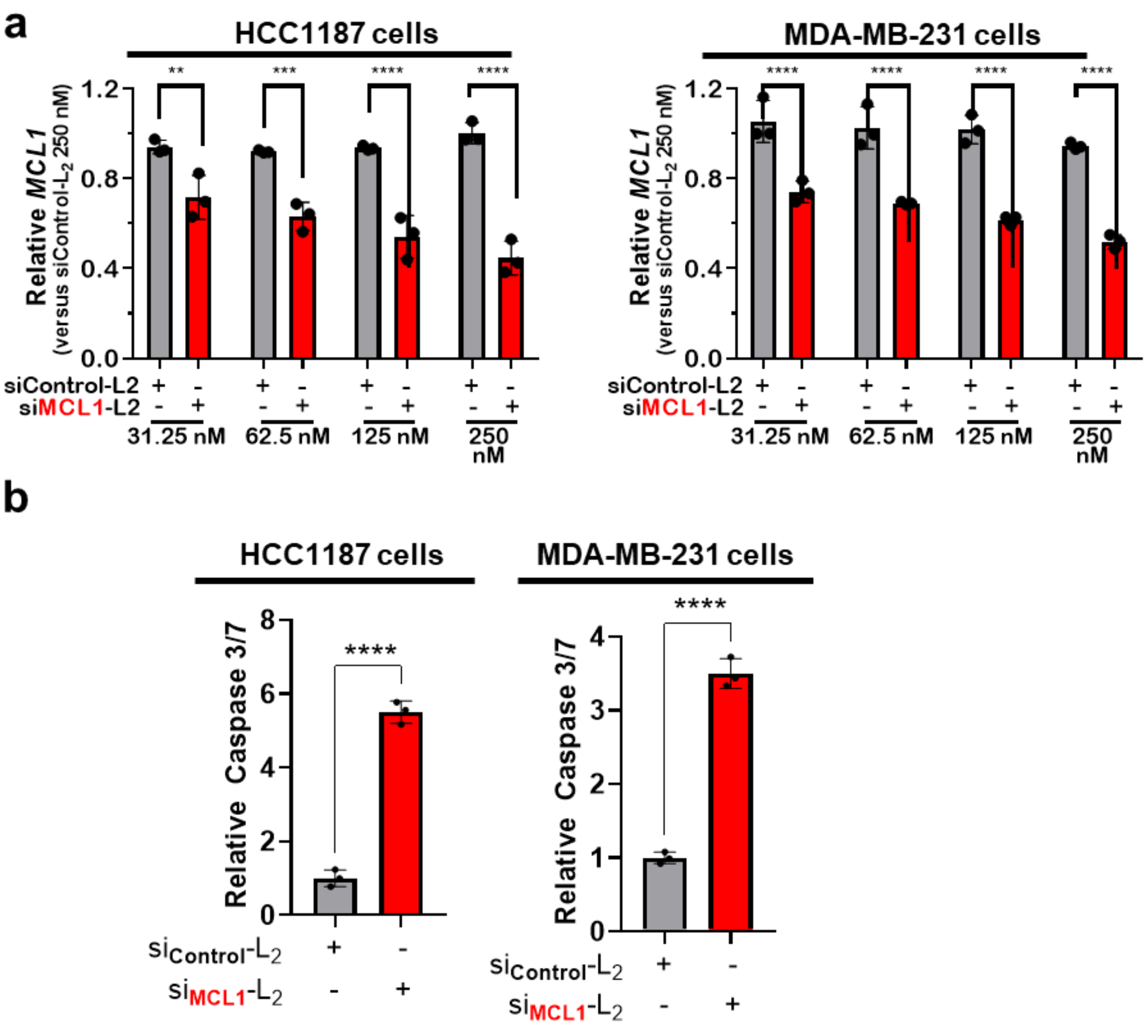

Supplementary Figure 19: (A) HCC1187 and MDA-MB-231 cells were treated in serum-free OptiMEM with siRNA-lipid conjugates and assessed 48 h post-treatment for expression of MCL1 transcript levels, corrected for expression of the housekeeping gene PPIB. Results are shown relative to *MCL1* levels seen in si$_{Control}$-L$_2$ (250 nM)-treated cells. Significance determined by 2-way ANOVA with Sidak's multiple comparisons test (n=3). (B) HCC1187 and MDA-MB-231 cells treated with siRNA-lipid conjugates for 96 h at 250 nM were assessed using Caspase 3/7-Glo. Significance determined by unpaired t-test (n=3).

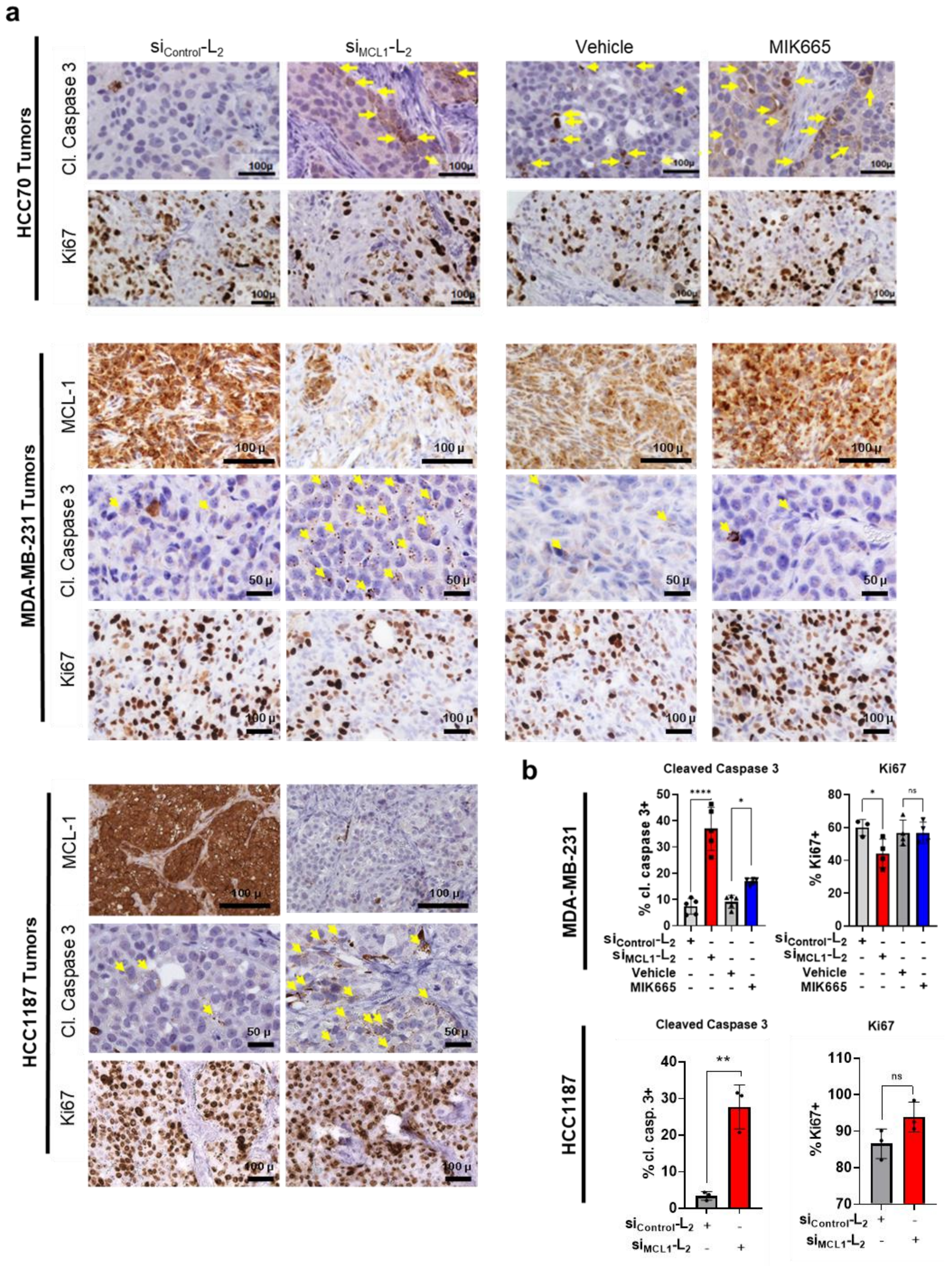

Supplementary Figure 20: (A) Mice bearing orthotopic HCC70, HCC1187, or MDA-MB-231 tumors were treated on day 0 and day 7 with si$_{Control}$-L$_2$ or si$_{MCL-1}$-L$_2$ (10 mg/kg, delivered i.v.). Tumors were collected on day

8. N = 3-4. Representative images of immunohistochemical staining for cleaved caspase 3, Ki67, and MCL-1 are shown. (B) Quantification of the percentage of HCC1187 and MDA-MB-231 tumor cells staining positive for Ki67 and cleaved (cl.) caspase is shown. (HCC70 quantitation is shown in Figure 9). MDA-MB-231 study results were analyzed for significance using 1-way ANOVA with Tukey's multiple comparisons test (n=3-5). HCC1187 study results were analyzed for significance using an unpaired t-test (n=3).

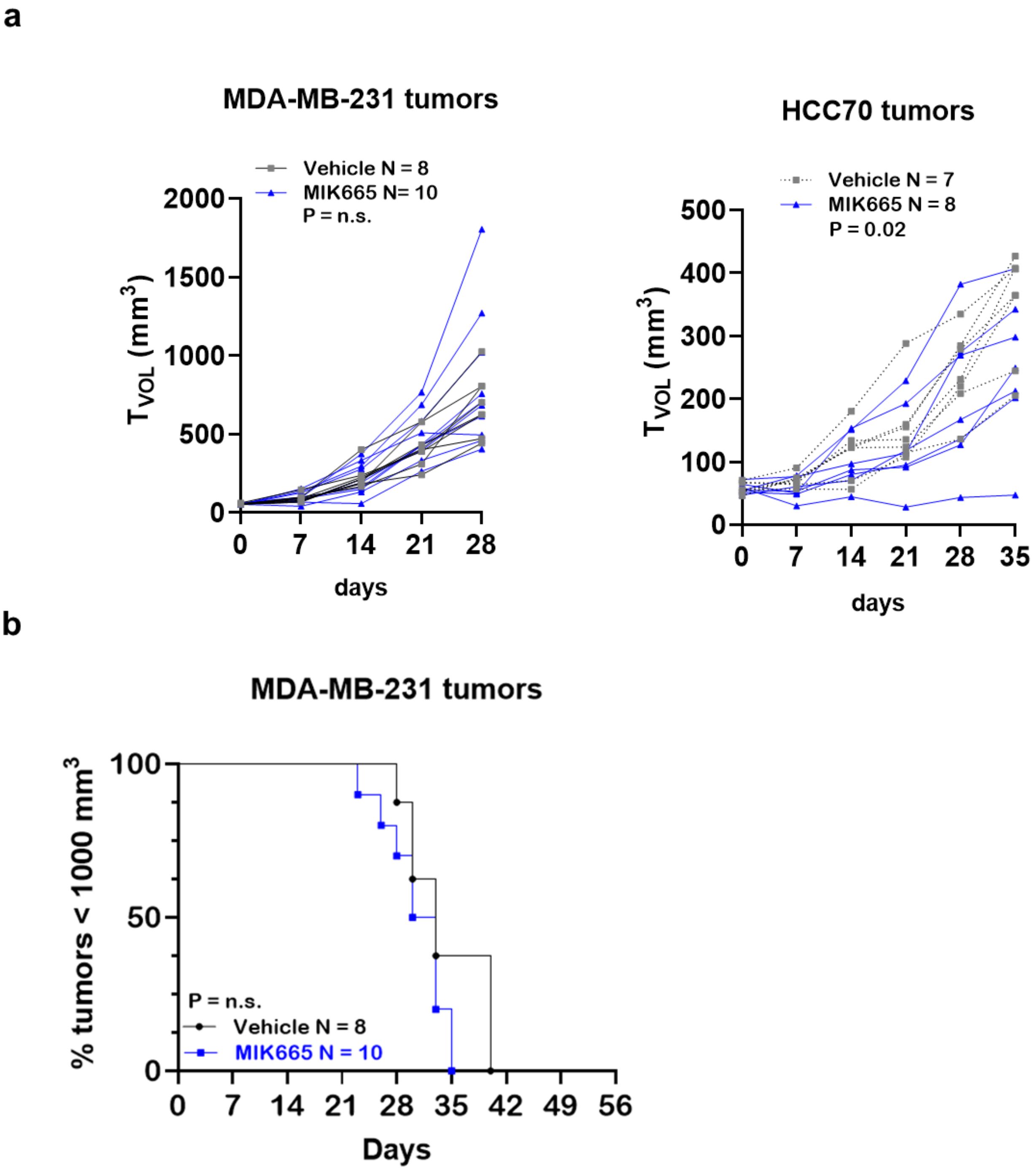

Supplementary Figure 21: Mice harboring HCC70 (N = 7-8) and MDA-MB-231 (N = 8-10) orthotopic tumors (50 -100 mm^3) received once weekly doses of vehicle (used for delivery of MIK665), or MIK665 through day 28 by i.v. injection. Tumors were collected on day 35 (HCC70) or day 56 (MDA-MB-231) or earlier if tumors ulcerated or exceeded humane size limitations. (A) Tumor volumes were measured throughout treatment. Statistical values were calculated based on area under the curve. (B) Kaplan-Meier analysis of tumor bearing

mice, defining survival as tumor volume under 1000 mm^3. P values are calculated using the log-rank (Mantel-Cox) test.
