# Supplementary material for: Structural optimization of siRNA conjugates for albumin binding achieves effective MCL1-directed cancer therapy
Source: Nat Commun. 2024 Feb 21;15:1581. doi: 10.1038/s41467-024-45609-0 (PMC10881965; doi:10.1038/s41467-024-45609-0)
Supplement: Supplementary file 3 — Reporting Summary [file 41467_2024_45609_MOESM3_ESM.pdf]

nature portfolio

Corresponding author(s): Craig Duvall

Last updated by author(s): Nov 28, 2023

Reporting Summary

Nature Portfolio wishes to improve the reproducibility of the work that we publish. This form provides structure for consistency and transparency in reporting. For further information on Nature Portfolio policies, see our Editorial Policies and the Editorial Policy Checklist.

Statistics

For all statistical analyses, confirm that the following items are present in the figure legend, table legend, main text, or Methods section.

n/a	Confirmed	
☐	☒	The exact sample size (*n*) for each experimental group/condition, given as a discrete number and unit of measurement
☐	☒	A statement on whether measurements were taken from distinct samples or whether the same sample was measured repeatedly
☐	☒	The statistical test(s) used AND whether they are one- or two-sided<br>*Only common tests should be described solely by name; describe more complex techniques in the Methods section.*
☒	☐	A description of all covariates tested
☐	☒	A description of any assumptions or corrections, such as tests of normality and adjustment for multiple comparisons
☐	☒	A full description of the statistical parameters including central tendency (e.g. means) or other basic estimates (e.g. regression coefficient) AND variation (e.g. standard deviation) or associated estimates of uncertainty (e.g. confidence intervals)
☒	☐	For null hypothesis testing, the test statistic (e.g. *F*, *t*, *r*) with confidence intervals, effect sizes, degrees of freedom and *P* value noted<br>*Give P values as exact values whenever suitable.*
☒	☐	For Bayesian analysis, information on the choice of priors and Markov chain Monte Carlo settings
☒	☐	For hierarchical and complex designs, identification of the appropriate level for tests and full reporting of outcomes
☒	☐	Estimates of effect sizes (e.g. Cohen's *d*, Pearson's *r*), indicating how they were calculated

Our web collection on statistics for biologists contains articles on many of the points above.

Software and code

Policy information about availability of computer code

Data collection	No code was used
Data analysis	No code was used

For manuscripts utilizing custom algorithms or software that are central to the research but not yet described in published literature, software must be made available to editors and reviewers. We strongly encourage code deposition in a community repository (e.g. GitHub). See the Nature Portfolio guidelines for submitting code & software for further information.

Data

Policy information about availability of data

All manuscripts must include a data availability statement. This statement should provide the following information, where applicable:

- Accession codes, unique identifiers, or web links for publicly available datasets
- A description of any restrictions on data availability
- For clinical datasets or third party data, please ensure that the statement adheres to our policy

The raw/processed data required to reproduce these findings are available upon request

Human research participants

Policy information about studies involving human research participants and Sex and Gender in Research.

Reporting on sex and gender	N/A
Population characteristics	N/A
Recruitment	N/A
Ethics oversight	N/A

Note that full information on the approval of the study protocol must also be provided in the manuscript.

Field-specific reporting

Please select the one below that is the best fit for your research. If you are not sure, read the appropriate sections before making your selection.

☒ Life sciences ☐ Behavioural & social sciences ☐ Ecological, evolutionary & environmental sciences

For a reference copy of the document with all sections, see nature.com/documents/nr-reporting-summary-flat.pdf

Life sciences study design

All studies must disclose on these points even when the disclosure is negative.

Sample size	Based on standard deviation of 18% estimated from preliminary data, n = 7 per tumor type per group was determined to provide 80% power to detect a 30% difference in change in tumor volume, p<0.05.
Data exclusions	No data were excluded from the study
Replication	In vivo pharmacokinetic samples were taken from multiple days and batches of formulations to ensure repeatability. To reduce the number of animals used, tumor-bearing mouse studies were not repeated beyond the number of mice per group in each study, as indicated.
Randomization	Tumor size was measured and used to equitably distribute a range of tumor sizes to each treatment group.
Blinding	Tumor measurement was performed blinded.

Reporting for specific materials, systems and methods

We require information from authors about some types of materials, experimental systems and methods used in many studies. Here, indicate whether each material, system or method listed is relevant to your study. If you are not sure if a list item applies to your research, read the appropriate section before selecting a response.

Materials & experimental systems

n/a	Involved in the study	
☐	☒	Antibodies
☐	☒	Eukaryotic cell lines
☒	☐	Palaeontology and archaeology
☐	☒	Animals and other organisms
☒	☐	Clinical data
☒	☐	Dual use research of concern

Methods

n/a	Involved in the study	
☒	☐	ChIP-seq
☐	☒	Flow cytometry
☒	☐	MRI-based neuroimaging

Antibodies

Antibodies used	PE anti-mouse CD19 antibody (Biolegend Catalog No. 115508), APC anti-mouse/human CD45R/B220 antibody (Biolegend Catalog No. 103212)
Validation	Both antibodies were validated by the manufacturer for use in flow cytometric analyses.

Eukaryotic cell lines

Policy information about cell lines and Sex and Gender in Research

Cell line source(s)	MDA-MB-231, HCC70, and HCC1187 cells were purchased from ATCC.
Authentication	Authentication performed by ATCC.
Mycoplasma contamination	Mycoplasma contamination testing was done by MycoAlert Myocplasma Detection Kit (Lonza).
Commonly misidentified lines (See ICLAC register)	N/A

Animals and other research organisms

Policy information about studies involving animals; ARRIVE guidelines recommended for reporting animal research, and Sex and Gender in Research

Laboratory animals	Pharmacokinetic studies were performed in 6-8 week-old male CD-1 mice (Charles River).<br>Toxicity studies in wild-type mice were performed in both male and female 4-6 week-old CD-1 mice (Charles River).<br>For tumor studies, 4-6 week-old female athymic nu/nu mice were used (Envigo).
Wild animals	N/A
Reporting on sex	For toxicity studies, both male and female mice were used to accurately capture the effects of treatment. Male mice were used for pharmacokinetic studies.<br>Because these studies are designed to interrogate efficacy in breast cancer, female mice were used for all tumor-bearing mouse models. Tumors were inoculated into the mammary of the female mice.
Field-collected samples	N/A
Ethics oversight	All animal experiments were IACUC approved and performed according to AAALAC guidelines and NIH best practices in AAALAC-accredited facilities at Vanderbilt University.

Note that full information on the approval of the study protocol must also be provided in the manuscript.

Flow Cytometry

Plots

Confirm that:

☒ The axis labels state the marker and fluorochrome used (e.g. CD4-FITC).

☒ The axis scales are clearly visible. Include numbers along axes only for bottom left plot of group (a 'group' is an analysis of identical markers).

☒ All plots are contour plots with outliers or pseudocolor plots.

☒ A numerical value for number of cells or percentage (with statistics) is provided.

Methodology

Sample preparation	For tumor cell uptake of Cy5-labeled siRNA-lipid conjugates, tumors were minced in HBSS (with Ca2+ and Mg2+), dissociated at 37ºC for 1h in collagenase (0.5 mg/mL, Roche Life Sciences) / DNase (0.19 mg/mL, BioRAD) in DMEM, and filtered through a 70 µm cell strainer. Erythrocytes were removed using ACK lysis buffer (Thermo Fisher Scientific) for 2 min.<br><br>For B cell analyses, spleens were pressed into PBS-/- and 1mM EDTA, passed through a 70 µM cell strainer, and washed. Excised femorae and tibiae were flushed with 10 mL of 1% fetal calf serum in EDTA (5 mM) + PBS-/- using a 25G needle. The exudate was filtered (70 µM) and washed. Erythrocytes were removed from splenocytes and bone marrow cells (BMCs) using ACK lysis buffer for 3 min. 1 x 10^6 cells/mL were blocked with mouse Fc block and then stained with the fluorophore-conjugated antibodies CD19-PE (1:200) and B200-APC (1:400).
Instrument	Dissociated tumor cells were assessed using a Guava easyCyte; B cells isolated from spleens and bone marrow were run on a BD FACS Diva.
Software	FlowJo software was used to analyze data.
Cell population abundance	See below.
Gating strategy	Tumors from mice treated with saline in lieu of a fluorescent article were used to determine thresholds for cells positive or negative for fluorescence.<br>For B cells isolated from spleens and bone marrow, gates were determined with fluorescence minus one (FMO) controls. B

March 2021

cells were defined as high intensity staining of CD19 PE and B200 APC. All plots used for compensation and gating are presented in the supplementary information.

☒ Tick this box to confirm that a figure exemplifying the gating strategy is provided in the Supplementary Information.
